# Multifaceted roles of LhWRKY44 in promoting anthocyanin accumulation in Asiatic hybrid lilies (*Lilium* spp.)

**DOI:** 10.1093/hr/uhad167

**Published:** 2023-08-22

**Authors:** Mengmeng Bi, Rui Liang, Jiawen Wang, Yuxiao Qu, Xin Liu, Yuwei Cao, Guoren He, Yue Yang, Panpan Yang, Leifeng Xu, Jun Ming

**Affiliations:** State Key Laboratory of Vegetable Biobreeding, Institute of Vegetables and Flowers, Chinese Academy of Agricultural Sciences, Beijing, 100081, China; State Key Laboratory of Vegetable Biobreeding, Institute of Vegetables and Flowers, Chinese Academy of Agricultural Sciences, Beijing, 100081, China; College of Horticulture, Shanxi Agricultural University, Taigu, 030031, China; State Key Laboratory of Vegetable Biobreeding, Institute of Vegetables and Flowers, Chinese Academy of Agricultural Sciences, Beijing, 100081, China; State Key Laboratory of Vegetable Biobreeding, Institute of Vegetables and Flowers, Chinese Academy of Agricultural Sciences, Beijing, 100081, China; State Key Laboratory of Vegetable Biobreeding, Institute of Vegetables and Flowers, Chinese Academy of Agricultural Sciences, Beijing, 100081, China; College of Landscape architecture and Forestry, Qingdao Agricultural University, Qingdao, 266109, China; State Key Laboratory of Vegetable Biobreeding, Institute of Vegetables and Flowers, Chinese Academy of Agricultural Sciences, Beijing, 100081, China; College of Chemistry and Life Science, Gannan Normal University, Ganzhou, 341000, China; State Key Laboratory of Vegetable Biobreeding, Institute of Vegetables and Flowers, Chinese Academy of Agricultural Sciences, Beijing, 100081, China; Shanghai Key Laboratory of Plant Molecular Science, College of Life Sciences, Shanghai Normal University, Shanghai, 200233, China; State Key Laboratory of Vegetable Biobreeding, Institute of Vegetables and Flowers, Chinese Academy of Agricultural Sciences, Beijing, 100081, China; State Key Laboratory of Vegetable Biobreeding, Institute of Vegetables and Flowers, Chinese Academy of Agricultural Sciences, Beijing, 100081, China; State Key Laboratory of Vegetable Biobreeding, Institute of Vegetables and Flowers, Chinese Academy of Agricultural Sciences, Beijing, 100081, China; State Key Laboratory of Vegetable Biobreeding, Institute of Vegetables and Flowers, Chinese Academy of Agricultural Sciences, Beijing, 100081, China

## Abstract

The Asiatic hybrid lily (*Lilium* spp.) is a horticultural crop with high commercial value and diverse anthocyanin pigmentation patterns. However, the regulatory mechanism underlying lily flower color has been largely unexplored. Here, we identified a WRKY transcription factor from lily tepals, LhWRKY44, whose expression was closely associated with anthocyanin accumulation. Functional verification indicated that LhWRKY44 positively regulated anthocyanin accumulation. LhWRKY44 physically interacted with LhMYBSPLATTER and directly bound to the *LhMYBSPLATTER* promoter, which enhanced the effect of the LhMYBSPLATTER-LhbHLH2 MBW complex activator on anthocyanin accumulation. Moreover, EMSA and dual-luciferase assays revealed that LhWRKY44 activated and bound to the promoters of gene *LhF3H* and the intracellular anthocyanin-related glutathione S-transferase gene *LhGST*. Interestingly, our further results showed that LhWRKY44 participated in light and drought-induced anthocyanin accumulation, and improved the drought tolerance in lily via activating stress-related genes*.* These results generated a multifaceted regulatory mechanism for the LhWRKY44-meditaed enhancement by the environmental signal pathway of anthocyanin accumulation and expanded our understanding of the WRKY-mediated transcriptional regulatory hierarchy modulating anthocyanin accumulation in Asiatic hybrid lilies.

## Introduction

Lilies are among the most common and popular ornamental plants grown worldwide and have diverse floral colors. Anthocyanins, which are among the major pigments responsible for the colors of lily tepals, belong to the flavonoid family of plant secondary metabolites and are mainly distributed in the vacuoles of plant tissues. In addition to determining the special colors of plants, these molecules act as protective substances that resist environmental stress, aid pollination and scavenge reactive oxygen species [1].

Anthocyanin biosynthesis is the most clearly studied metabolic pathway and involves a series of catalytic enzymes encoded by genes needed for synthesis. These enzymes include the production of common precursor genes (*PAL*, *CHI*, *CHS*, *F3H*, and *F3’H*) and late biosynthetic genes (*DFR*, *AN*S, *UFGT*, etc.) [2]. Anthocyanins are catalyzed in the cytoplasm and transported to vacuoles with the of assistance transport proteins. Therefore, some molecular carriers can mobilize and transport anthocyanins from the cytoplasm to vacuoles during anthocyanin synthesis [3]. It has been suggested that GST family genes serve as regulators of anthocyanin sequestration and transport in several plants, including *Perilla frutescens*, *Cyclamen persicum*, *Dianthus caryophyllus* and *Lily*[4–7]. The accumulation of anthocyanins is mostly regulated at the transcriptional level by the conserved MBW complex composed of R2R3-MYB, bHLH, and WD40 proteins [8]. In lily cultivars, various and complex floral pigmentation patterns are observed, and these include bicolor, splatter-type spots, raised spots, and blush marks in addition to a wide color variation [9]. Numerous R2R3-MYB proteins involved in anthocyanin biosynthesis have been characterized. Different R2R3-MYB genes are responsible for lily coloration in different genetic backgrounds. In the Oriental hybrid lily, LhMYB12 often regulates the color formation of whole-pink-colored tepals and induces spot pigmentation [10–12]. In Trumpet hybrid lilies, LrMYB15 regulates the coloring of the lower epidermis of the tepals of *Lilium regale* [13]. In the Tango series cultivars of the Asiatic hybrid lily, LhMYBSPLATTER determines the formation of splashy spots caused by anthocyanin in tepals [14–16]. Additionally, LhbHLH2 plays a role in anthocyanin accumulation in Asiatic hybrid lily [10, 17]. The WD repeat gene *LhWDR* interacts with LhbHLH2 to co-regulate anthocyanin accumulation in lily [18].

In addition to the MBW complex, TFs such as SPL, NAC, AP2/ERF, HD-Zip, BBX, HY5, and WRKY were recently found to regulate anthocyanin accumulation in plants. These TFs function in anthocyanin accumulation by directly or indirectly regulating structural genes or the MBW complex [19, 20]. WRKY superfamily members are characterized by the conserved WRKYGQK domain and zinc-finger motif. They can recognize and bind to a specific W-box sequence (C/T) TGAC (T/C) to play regulatory roles [21]. Previous studies have extensively studied the critical role of WRKY TFs in plant stress, flowering, development and other processes. Recently, more studies have focused on WRKYs, which participate in flavonoid synthesis-associated processes in plants. These genes include *AtWRKY75*, *MdWRKY11*, *MdWRKY40,* and *PyWRKY26*[22–25]. However, anthocyanin-related WRKYs from eudicots and monocots may have diverged before they evolved into specialized angiosperm WRKYs. Functional and mechanistic differentiation of WRKYs exists in different plants. In *Arabidopsis*, a gene encodes a WRKY44 TF, *TTG2*, which determines trichome formation and seed coat development in the PA pathway [26]. *Brassica napus* TTG2 suppresses auxin biosynthesis gene expression to increase salt stress sensitivity [27]. In *Petunia*, the PH3 TF, which is highly similar to AtTTG2, regulates flavonoid biosynthesis by acidifying the vacuole [28]. In *Hylocereus polyrhizus*, HpWRKY44 contributes to betalain biosynthesis by regulating *HpCytP450-like1*, which contributes to betalain biosynthesis [29]. In pear, PpWRKY44 transcriptionally activates *PpMYB10* expression to accelerate anthocyanin accumulation [30]. Although some evidences have shown that WRKY TFs regulate anthocyanin accumulation in eudicots plants, the involvement of WRKY TFs in anthocyanin biosynthesis in monocot plants, particularly in lily has been largely unexplored. No documented data have implicated the mechanism mediated by WRKY TFs in anthocyanin accumulation in lily flowers.

Tango series cultivars are a type of Asiatic hybrid lily and frequently develop red spots on the interior surfaces of their tepals, and anthocyanin accumulation within these spots appears on the flowers. Many small splatter spots (speckles) develop in the lower half of the tepals [9, 31]. In the current study, we isolated and identified the positive roles of LhWRKY44 (c117585_—_g2) on anthocyanin accumulation in lily based on previously obtained RNA-seq (SRP093907) data [15]. The multifaceted regulation of LhWRKY44 on lily anthocyain pigment formation was further revealed. Our findings clarify the mechanism of anthocyanin accumulation in Asiatic hybrid lilies and enrich the effects of the TF transcriptional regulation of anthocyanins.

## Results

### LhWRKY44 is a transcription factor related to anthocyanin biosynthesis

**Figure 1:**
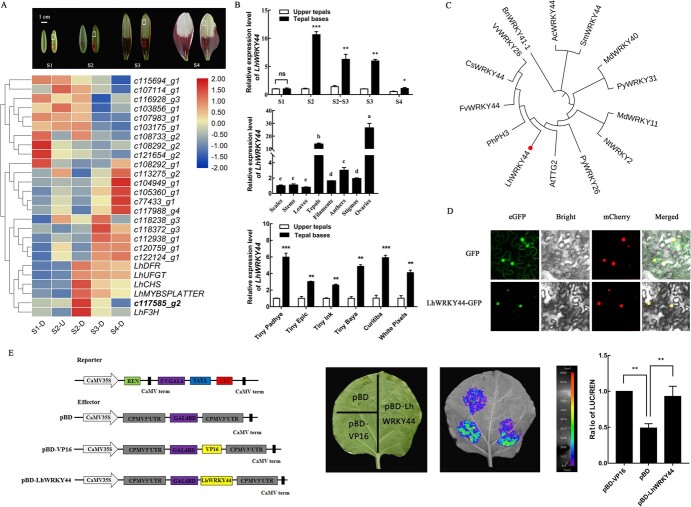
Characterization of the LhWRKY44 transcription factor. **A** Heat map of anthocyain biosynthesis-related genes and candidate WRKY genes expression at tepal different development stages of the Asiatic hybrid lily cultivar ‘Tiny Padhye’. The gene (c117585_g2) with bold font is predicted *LhWRKY44* gene. Different development stages (S1, S2, S3, and S4) used for RNA-seq. The flower buds’ development stages (S) are as follow: Stage 1 (S1: bud length of 2 cm, no anthocyanins visible on tepals); Stage 2 (S2: anthocyanins visible on tepal bases); Stage 2–Stage 3 (S2–S3: anthocyanins visible on half of the tepals); Stage 3 (S3: 1 d before anthesis, where the lower half of the tepals were fully pigmented); and Stage 4 (S4: 0 d post-anthesis). Colored boxes indicate tepal parts used for experiments (white boxes, U: upper tepals; red boxes, D: basal tepals). Samples were collected from 20 tepals as one biological sample, the upper tepals and bases of inner tepals were collected separately. Scale bar,1 cm. **B** Expression of *LhWRKY44* at different developmental stages and different tissues of lily. Expression of *LhWRKY44* in six Tango series lily cultivars. Flowers at the same stage were subjected to RNA extraction. The lily *actin* gene was used as the control. **C** Phylogenetic analysis of LhWRKY44 and other anthocyanin-related WRKYs. The amino acid sequences of SmWRKY44 (AKA27910), PhPH3 (AMR43368), AtTTG2 (NP_181263), CsWRKY44 (AYA73391), VvWRKY26 (AQM37647), BnWRKY41–1 (XP_013686534), PyWRKY26 (Pbr013092), PyWRKY31 (Pbr000122), FvWRKY44 (XM_004302784), NtWRKY2 (AB_063576), MdWRKY11 (MDP0000128463), AcWRKY44 (ACC16887), and MdWRKY40 (XP_008342807) were retrieved from the GenBank database. **D** Subcellular localization of LhWRKY44-GFP. eGFP, GFP signal; mCherry, nuclear marker; Bright, white light; Merged, combination of the GFP, bright field, and mCherry signals. Scale bars, 25 μm. **E** Transcriptional activity analysis of LhWRKY44 in *Nicotiana benthamian*a leaves. Schematic diagram of vectors (left). pBD-LhWRKY44, the recombinant plasmid containing LhWRKY44; pBD, the negative control; pBD-VP16, the positive control. Live imaging and quantitative analysis (right).

On the basis of transcriptome data, we screened a differentially expressed WRKY gene, *LhWRKY44* (Fig. 1A). To explore the expression pattern of *LhWRKY44*, we analysed the transcript levels of *LhWRKY44* at different developmental stages and in different tissues by RT–qPCR. The results showed that *LhWRKY44* was always higher in basal tepals than in upper tepals, in accordance with the total anthocyanin and anthocyanin biosynthesis pathway genes level. The tissue-specific expression pattern analysis revealed that the expression level of *LhWRKY44* in different tissues were higher in the flower and ovary tissues than in the anthers, filaments, stigmas, stems, scales, and leaves (Fig. 1A and B). To further confirm that the expression of *LhWRKY44* is related to anthocyanin biosynthesis, five other Tango series cultivars were chosen to determine the expression of *LhWRKY44* in basal (high anthocyanin level) and upper (low anthocyanin level) tepals at the same developmental stage. The result demonstrated that the level of *LhWRKY44* was always higher in basal tepals than in upper tepals, consistent with the anthocyanin content and the level of *LhCHI*, *LhF3H*, *LhMYBSPLATTER*, etc. anthocyanin biosynthesis-related genes (Fig. 1B; Fig. S1, see online supplementary material). These data suggested that *LhWRKY44* may be related to anthocyanin accumulation in lily tepals.

Full-length 1972-bp LhWRKY44 cDNA was isolated from ‘Tiny Padhye’ tepals by RACE. The segment comprised 1386-bp open reading frames encoding a 461-residue polypeptide. A sequence analysis showed that the LhWRKY44 protein harbored two conserved WRKYGQK domains toward the N-terminus and a zinc finger ligand (Fig. S2A, see online supplementary material). Cluster analysis indicated that LhWRKY44 was most closely related to a branch containing AtWRKY44 (TTG2). Hence, it was designated LhWRKY44 (Fig. S2B, see online supplementary material). LhWRKY44 belonged to group I of the WRKY superfamily. A phylogenetic analysis of WRKY proteins that had been confirmed to be associated with anthocyanin accumulation in multiple species showed that LhWRKY44 shares a close evolutionary relationship with PhPH3 (WRKY44) of *Petunia* and AtTTG2 of *Arabidopsis* (Fig. 1C). To confirm the putative function of LhWRKY44 as a TF, we generated a LhWRKY44-GFP recombinant protein and conducted a subcellular localization assay. The fluorescence signal matched the GFP and mCherry signal (Fig. 1D), indicating that LhWRKY44 localizes to the nucleus. Further analysis of the transcriptional activity in *Nicotiana benthamiana* leaf cells revealed that LhWRKY44 had transcriptional activation ability. Similar to the positive control pBD-VP16, the relative luciferase of pBD-LhWRKY44 exhibited markedly higher activity than the negative control pBD (Fig. 1E). Therefore, LhWRKY44 might work as a nucleus-localized transcriptional activator related to anthocyanin biosynthesis.

### LhWRKY44 promotes anthocyanin accumulation in lily

To study whether LhWRKY44 plays a role in anthocyanin accumulation, a transient transformation assay involving *Nicotiana tabacum* leaves was conducted as previously described [32]. The results showed that *LhWRKY44* induced anthocyanin accumulation in tobacco leaves (Fig. ure S3A, see online supplementary material). Because stable genetic transformation has not been well established in lilies, it remains difficult to obtain stable genetic plants by Agrobacterium. Thus, we ectopically stable overexpressed *LhWRKY44* in ‘Orin’ apple calli and generated transgenic lines. An obvious color change and markedly higher total anthocyanin content were observed in the transgenic lines of *LhWRKY44* compared with the WT and control. qPCR analysis showed that these genes were anthocyanin pathway structural genes also up-regulated after *LhWRKY44* overexpression (Fig. S3B, see online supplementary material). Thus, overexpression of *LhWRKY44* facilitated anthocyanin accumulation.

**Figure 2:**
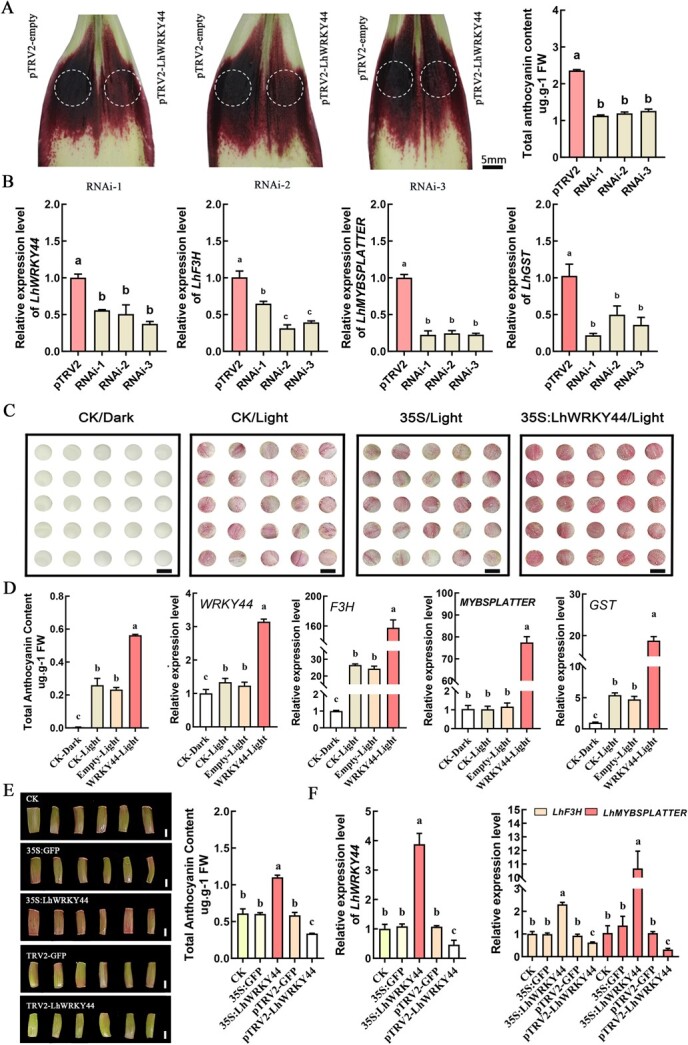
Functional analysis of *LhWRKY44* in anthocyanin accumulation of lily. **A** Transient silence of *LhWRKY44* gene repressed anthocyanin accumulation in lily tepals. Diagrams of sampling positions in the infiltrated sites of silenced lily tepals and total anthocyanin content. Phenotypic images were taken 7 d after infiltration. Representative pictures of three lines were shown. Bar, 5 mm. **B** Expression level of *LhWRKY44* and anthocyanin accumulation-related genes after silencing. **C** Transient overexpression of *LhWRKY44* gene promoted anthocyanin accumulation in lily scales. Bar, 1 cm. **D** Total anthocyanin and level of *LhWRKY44* and anthocyanin pathway genes after overexpressing. **E** Phenotype observation of lily leaves after overexpressing and silencing *LhWRKY44* gene. Bar, 1 cm. **F** Expression level of *LhWRKY44* and anthocyanin accumulation-related genes.

To further investigate the function of LhWRKY44 in lily anthocyanin accumulation, previous transient transformation experiments in lily were carried out [33]. Agrobacterium cultures harboring pTRV2 and pTRV2-LhWRKY44 were used to infiltrate the outer tepals of lily cultivar ‘Tiny Padhye’ buds. When tepals from lily buds at the same stage were infected with the recombinant TRV construct, the color of those infected with the pTRV2-LhWRKY44 vector lines was clearly lighter than that of the empty control (Fig. 2A). The expression of *LhWRKY44* was successfully inhibited. The silencing of *LhWRKY44* also had weaken the *LhF3H* and *LhGST* transcript level, especially *LhMYBSPLATTER* (Fig. 2B). Meanwhile, a transient transform assay was performed in lily scales and leaves by a vacuum penetration method [34, 35]. Lily scales are usually could slightly accumulate anthocyanintransferred to light condition. When under continuous white light but not under dark conditions, a small amount of anthocyanin was formed after 4 d in the control treated with water (CK) or empty control. However, the presence of LhWRKY44 caused a deeper color than that observed in the control (Fig. 2C). As expected, further qPCR showed that LhWRKY44 also activated the level of *LhF3H*, *LhMYBSPLATTER*, and *LhGST* genes. Light promoted anthocyanin formation in lily, and it was significantly enhanced in the presence of LhWRKY44 (Fig. 2D). Results of function analysis in lily leaves further indicated that LhWRKY44 positively regulated anthocyanin accumulation (Fig. 2E and F).

**Figure 3:**
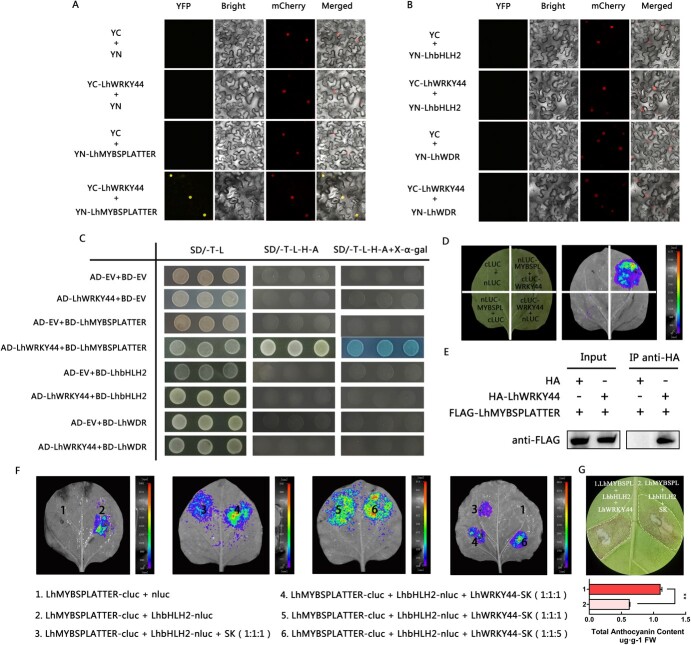
LhWRKY44 interacts with LhMYBSPLATTER and enhances the interaction between LhMYBSPLATTER and LhbHLH2. **A** and **B** BiFC assays of LhWRKY44 and the MBW complex in *Nicotiana benthamiana* leaves. **C** Yeast two-hybrid assay. **D** FLC assay. LUC signals were imaged by a CCD camera. **E** Coimmunoprecipitation assay. Input: total protein extracts. IP anti-HA: immunoprecipitation product with anti-HA antibody. Anti-FLAG: Immunoblots with anti-FLAG antibody. **F** Effects of LhWRKY44 on the interaction between LhMYBSPLATTER and LhbHLH2. FLC imaging assays. A schematic diagram of tobacco leaf injection is shown, and luciferase activity was captured by a CDD camera. **G** Transient co-infiltration *LhWRKY44*, *LhMYBSPLATTER*, and *LhbHLH2* in *Nicotiana tabacum* leaves.

### LhWRKY44 interacts with LhMYBSPLATTER

Many TFs interact with MBW complexes or function upstream to regulate anthocyanin accumulation [36]. Previously, we identified LhMYBSPLATTER, LhbHLH2 and LhWDR as potential LhWRKY44 interaction partners in Tango series lily cultivars ‘Tiny Padhye’ (Fig. 3G; Fig. S4, see online supplementary material). To check whether LhWRKY44 regulated anthocyanin biosynthesis via the MBW complex, directed BiFC assays were performed. The results confirmed that LhWRKY44 interacted with LhMYBSPLATTER but not with LhbHLH2 or LhWDR *in vivo* (Fig. 3A and B). In tobacco leaves with LhWRKY44-YFPC and LhMYBSPLATTER-YFPN, the fluorescence signal generated after the interaction of LhWRKY44 with LhMYBSPLATTER in the nucleus. A lack of fluorescence was observed upon coinfiltration with LhWRKY44-YFPC and YFPN or YFPC and LhMYBSPLATTER-YFPN and after infiltration of the combinations of LhWRKY44 with LhbHLH2 and LhWRKY44 with LhWDR. Subsequently, the interaction relationship was tested by Y2H assay and FLC imaging assay and the results supported the above-mentioned results (Fig. 3C and D). Co-IP assay showed the protein was successfully expressed in the input. LhWRKY44 protein was detected in the immune complex of LhMYBSPLATTER protein after enrichment by anti-HA agarose immunoprecipitation, whereas no obvious band could be observed in the negative control (empty), indicating LhWRKY44 interacted with LhMYBSPLATTER *in vivo* (Fig. 3E).

To examine the effect of LhWRKY44 on the MBW complex, we conducted an FLC imaging assay. LhMYBSPLATTER and LhbHLH2 fused to the N and C termini of luciferase, respectively. LhWRKY44 was inserted into the pGreenII 0029 62-SK vector. Tobacco leaves were coinfiltrated with different combinations of recombination plasmids. As shown in Fig. 3F, luminescence was observed in regions containing LhMYBSPLATTER-cluc and LhbHLH2-nluc but not in regions containing LhMYBSPLATTER-cluc and empty nluc. The coinfiltration of LhWRKY44-SK with LhMYBSPLATTER-cluc and LhbHLH2-nluc at a 1:1:1 ratio enhanced the luminescence signal, and the coinfiltration of LhWRKY44-SK with LhMYBSPLATTER-cluc and LhbHLH2-nluc at a 5:1:1 ratio strengthened the luminescence. LhWRKY44 enhanced the interaction between LhMYBSPLATTER and LhbHLH2 and promoted the formation of the MBW complex. Besides, a transient transformation assay with *N. tabacum* leaves was also conducted. As shown in Fig. 3G, the combination of LhMYBSPLATTER and LhbHLH2 triggered a stronger anthocyanin accumulation in the presence of LhWRKY44 compared with that obtained in the control (SK empty vector). The results clearly demonstrated that the interactions between LhWRKY44 and LhMYBSPLATTER promoted the formation and interaction of the MBW complex. The activated formation of the MBW complex increased anthocyanin accumulation and transcriptional activation in cells.

Notably, LhWRKY44 activated the expression of *LhMYBSPLATTER* (Fig. 2F; Fig. S5A and B, see online supplementary material). One specific W-box sequence was found on the promoter of *LhMYBSPLATTER.* Further assays indicated that LhWRKY44 bound to the W-box motif of the *LhMYBSPLATTER* promoter (Fig. S5C and D, see online supplementary material). Taken together, these results revealed a novel dual role for LhWRKY44-LhMYBSPLATTER in controlling anthocyanin accumulation in lily.

### LhWRKY44 directly binds to *LhF3H* and glutathione S-transferase gene *LhGST* promoters

**Figure 4:**
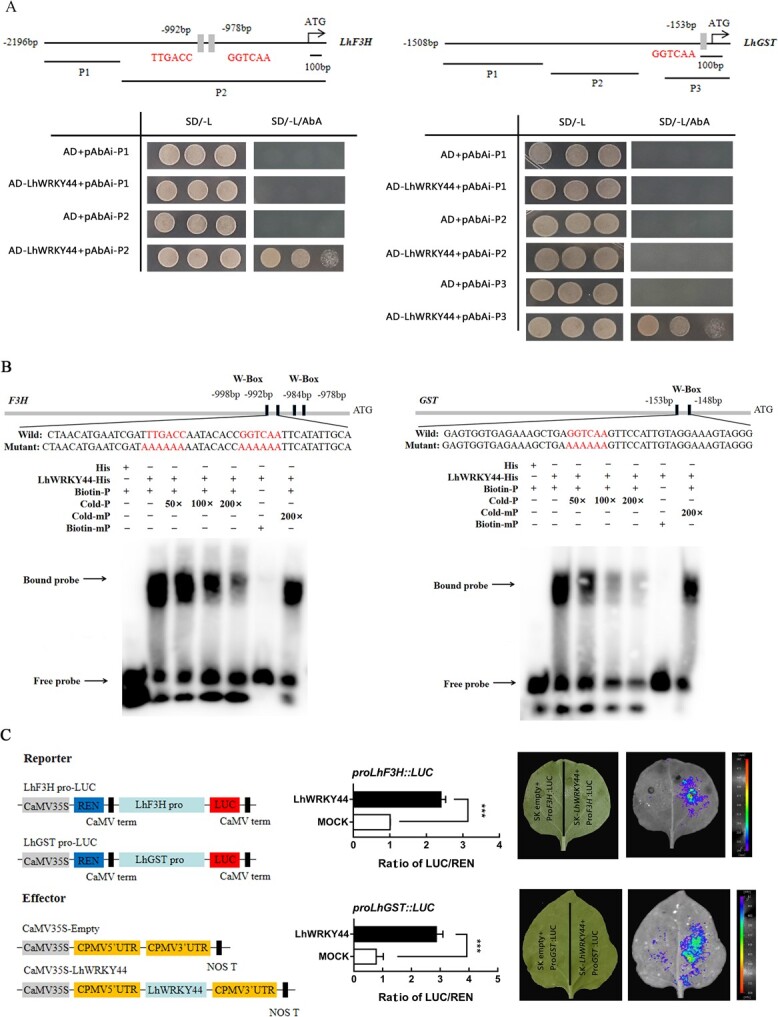
LhWRKY44 binds to *LhF3H* and *LhGST* promoters and activates their expression. **A** Y1H assay of the interaction between LhWRKY44 and *LhF3H* and *LhGST* promoters. **B** LhWRKY44 binds to the W-box elements of *LhF3H* and *LhGST* promoters in EMSAs. The probe sequences are shown. The term 200× indicates the usage of excess cold competition probe, and ‘+’ and ‘−‘ indicate its presence and absence, respectively. The arrows represent protein−DNA complexes or free probes. **C** Dual-luciferase assays. Vector diagrams, schematic diagram of the injected *Nicotiana benthamiana* leaves and living images by CDD were shown. LUC/REN ratio. SK(MOCK) represents the empty vector and is set to 1.

Because the anthocyanin pathway structural genes level were significantly down-regulated after LhWRKY44 silencing, these genes may be direct targets of LhWRKY44. To further explore the mechanism mediated by LhWRKY44 on anthocyanin accumulation, we performed Y1H assays. The basal activity of promoters was detected in yeast in the presence of aureobasidin A (AbA). The bait yeast cells co-transformed with LhWRKY44 and the negative control all survived on SD/−Leu. As shown in Fig. 4A, the yeast cells could survive on SD/−Leu/AbA when cotransformed with the *LhF3H* promoter but not with *LhCHS, LhDFR,* and *LhUFGT* promoters (Fig. S6, see online supplementary material). Besides, the promoter sequences showed the presence of at least one W-box motif in the *LhF3H* promoter. Electrophoresis mobility shift assays (EMSAs) were conducted, and the specific binding of LhWRKY44 to the W-box were characterized in *LhF3H* promoter. As shown in Fig. 4B, a mobility shift was observed when LhWRKY44-His protein was incubated with the W-box probe, and the shift was impaired after the addition of unlabeled competitor DNA probe but recovered in the presence of mutated unlabeled DNA probe. LhWRKY44 specifically bound to the promoter regions of *LhF3H*. Dual-luciferase assay between LhWRKY44 and anthocyain pathway structure genes showed LhWRKY44 activated the *LhF3H* promoter expression (Fig. 4C).

Anthocyanin is catalyzed by multiple enzymes in the cytoplasm and transported into the vacuole with the assistance of transport proteins. We previously demonstrated that a glutathione S-transferase gene, *LhGST*, was engaged in the anthocyanin transport process in Asiatic hybrid lilies [7]. Interestingly, we noted that the *LhGST* expression level was also affected by *LhWRKY44* (Fig. 2). The results from the dual-luciferase assay implied that LhWRKY44 activated the *LhGST* promoter (Fig. 4C). Additionally, one W-box motif was found within the region upstream of the start codon of the *LhGST* promoter. In the Y1H assay, three truncated regions of the *LhGST* promoter, namely, fragments P1, P2, and P3 were used as baits and assayed with the LhWRKY44 protein. LhWRKY44 bound to P1 containing the W-box motif and did not bind to P2 or P3 without the W-box (Fig. 4A). The EMSA result supported the above conclusion (Fig. 4B). Consequently, these results provide evidence showing that LhWRKY44 participates in the anthocyanin transport process in Asiatic hybrid lilies by directly targeting the *LhGST* promoter.

### *LhWRKY44* responses to light- and drought-induced anthocyanin biosynthesis

**Figure 5:**
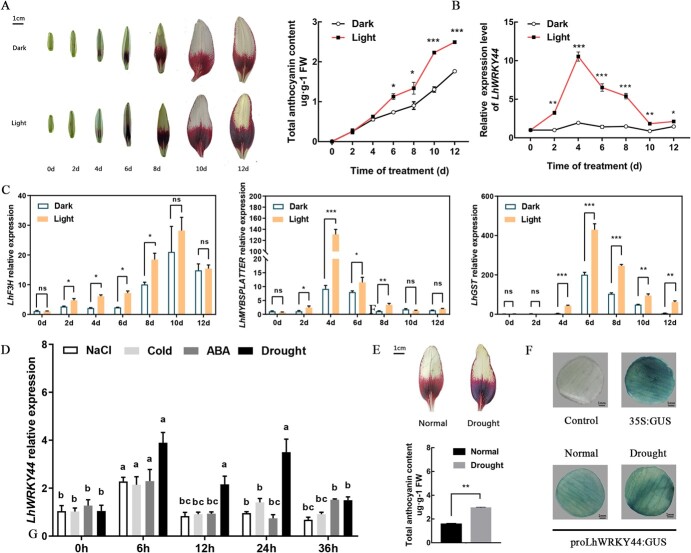
The expression of *LhWRKY44* is induced by light and drought treatment. Light treatment promotes lily flowers anthocyanin accumulation. Phenotype of lily flower buds were shown under dark and light treatment for different time. All samples are collected from the basal tepals. Scale bar, 1 cm. **B** The expression of *LhWRKY44* under dark and light treatment. **C** Expression level of anthocyanin synthesis-related after treatment. **D***LhWRKY44* expression analysis of drought, ABA, NaCl, and 4°C conditions. **E** Drought treatment for two weeks promotes lily flowers anthocyanin accumulation. **F** Drought treatment activates the GUS expression of *LhWRKY44* promoter in lily tepals*.*

To further reveal the underlying mechanism on anthocyanin accumulation regulated by *LhWRKY44*, we obtained the promoter of *LhWRKY44* according to chromosome walking. Sequence analysis showed that there are some light-responsive elements, such as GT1-motif, Sp1, MRE, LAMP-element, Gap-box, and TCT-motif, in addition to the TATA box and CAAT box core element, and stress response element, MBS, ABRE etc. (Table S1, see online supplementary material), thus we speculated that the expression of *LhWRKY44* may be induced by light or stress condition. Lily plants were subjected to high light and dark treatment. As shown as Fig. 5A, the anthocyanin accumulation significantly differed between the light-treated and control groups only after 6 d of the treatment period when flower buds were ready to open, and increasing light treatment times were associated with increasing intensity of the red coloration of lily tepals. The transcript levels of LhWRKY44 and anthocyanin pathway genes were highly up-regulated compared with control after exposure to light and peaked 4 d after treatment (Fig. 5B and C).

Considering that WRKY TFs always play a vital role in plants’ stress resisting process, we further analysed *LhWRKY44* expression following application of abiotic stress in lily and observed an ~4.0-fold increase in *LhWRKY44* transcript levels after 6 h and 24 h of drought treatment and an ~2-fold increase in *LhWRKY44* transcript levels after 12 h of drought treatment, while NaCl, ABA or 4°C resulted in more modest increases (2.6-fold for NaCl, 2.5-fold for 4°C and 2.4-fold for ABA) (Fig. 5D). Additionally, drought treatment also promoted anthocyanin accumulation in lily (Fig. 5E). GUS assay of the lily petal discs indicated that drought stress could activate the activity of *LhWRKY44* promoter in lily (Fig. 5F).

### Overexpression of *LhWRKY44* improves drought tolerance of transgenic *Arabidopsis* plants

**Figure 6:**
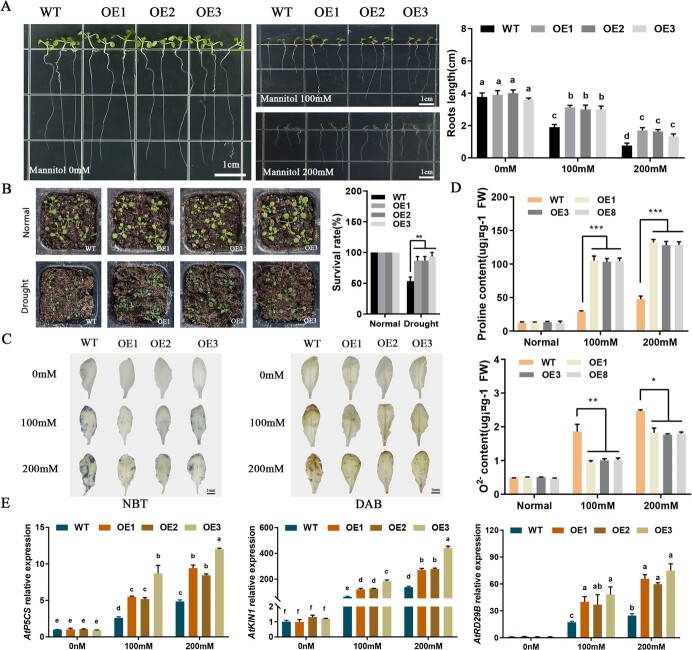
*LhWRKY44* transgenic *Arabidopsis* improves the tolerance to drought stress. **A** The root growth and length of WT and transgenic Arabidopsis lines cultured on 1/2 MS medium with 100 mM or 200 mM mannitol. Bars, 1 cm. **B** The seedlings growth phenotype and survival rate of WT and transgenic lines under drought treatment. **C** NBT and DAB staining of WT and transgenic lines treated with water and drought treatment. Bars,1 mm. **D** Proline and O^2−^ contents before and after mannitol treatment. **E** Expression of stress-related genes in WT and transgenic lines under mannitol stress.

To clarify the function of resisting drought stress by LhWRKY44, mannitol treatment was applied to WT Arabidopsis and the three homozygous LhWRKY44 transgenic lines (OE1, OE2, OE3). When grown on 1/2 MS medium, the WT and transgenic lines showed the similar phenotype in root length. However, the root length of the transgenic seedings showed a significant difference compared with WT plants under 100 mM mannitol medium, longer than that of the WT plants. Root growth remained superior in transgenic seedlings with the mannitol concentration increased to 200 mM (Fig. 6A). For the further analysis of drought stress tolerance, all seedlings were growth in soil treated with drought conditions for 20 days. There were no differences in phenotypic and plant survival rate between them under normal conditions, while the transgenic plant survival rate was significantly higher than that of WT plants under the same drought treatment (Fig. 6B). In addition, histochemical staining assay with NBT and DAB showed that the WT and transgenic plants had no difference, visualized as blue leaves and yellow, respectively, under normal conditions. With the increasing concentration of mannitol, the degree of blue staining of NBT products gradually deepens, and WT showed a higher degree than that of transgenic lines under the same mannitol treatment. Similarly, yellow staining of DAB products of WT was deepen than that of transgenic lines under the same mannitol treatment (Fig. 6C). Quantitation determination of the ROS level showed that the O^2−^content of the transgenic Arabidopsis lines was lower than that of WT under the same mannitol condition. The proline content was increased after *LhWRKY4*4 overexpression (Fig. 6D). Moreover, the expression of stress-related genes was up-regulated after overexpressing *LhWRKY44*. Under mannitol stress, the genes *AtKIN1*, *AtRD29B*, and *AtP5CS* all increased after overexpressing *LhWRKY44* (Fig. 6E).

As a result, overexpression of *LhWRKY44* enhanced tolerance to drought stress in *Arabidopsis*.

### LhWRKY44 confers the drought tolerance of lily by activating stress-related genes to promote anthocyanin accumulation

**Figure 7:**
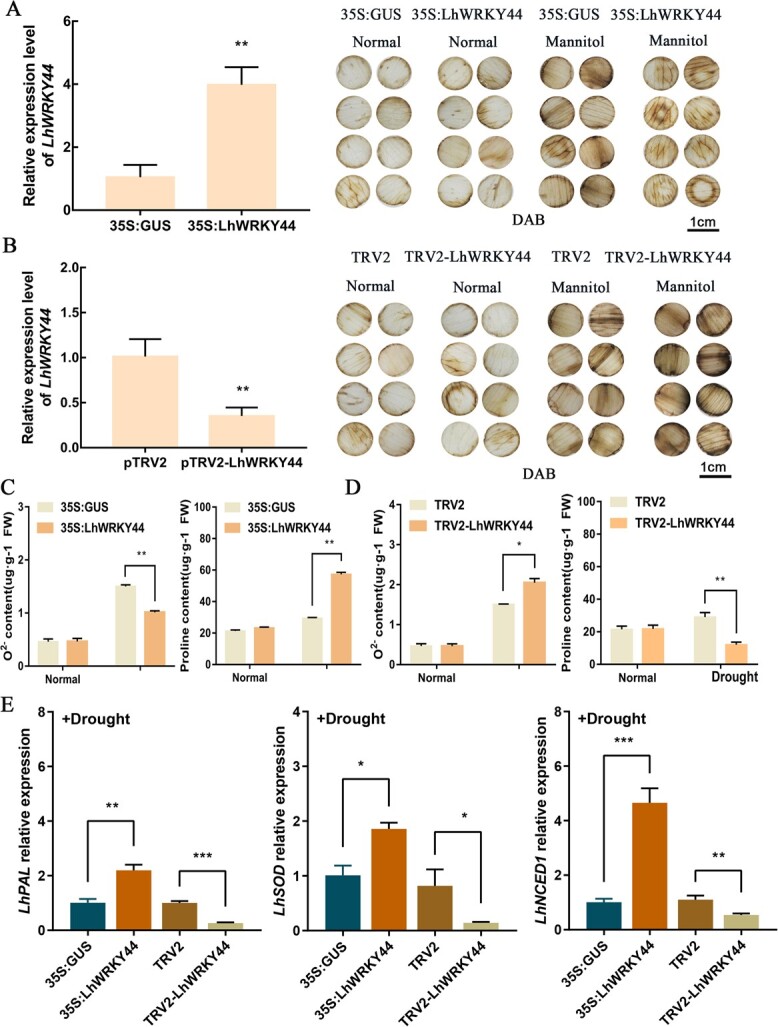
LhWRKY44 enhanced drought tolerance in lily via activating stress-related genes. **A** The *LhWRKY44* expression and DAB staining phenotypes in LhWRKY44-overexpression tepal discs under normal condition and drought stress (10% PEG6000). **B** The *LhWRKY44* expression and DAB staining phenotypes phenotypes in LhWRKY44-silence tepal discs under normal and drought stress. Representative image were shown. Scale bar, 1 cm. **C** O^2−^ and proline contents of LhWRKY44-overexpression lily tepal discs under normal and drought condition. **D** O^2−^and proline contents of LhWRKY44-silence lily tepal discs under normal and drought condition. **E** Relative level of stress-related genes of lily after drought treatment.

Considering drought treatment promotes lily flowers anthocyanin accumulation, we speculate LhWRKY44 might promote anthocyanin accumulation via drought abiotic stress pathways. Then, we overexpressed and silenced *LhWRKY44* in lily tepals via transient transformation [37] (Fig. 7A and B). There is no difference in the relative O^2−^ and proline content between LhWRKY44-overexpression discs and control discs, while the values for O^2−^ parameters were significantly higher in control discs than in LhWRKY44-overexpression discs after drought treatment. *LhWRKY44* overexpression improves the drought tolerance and increased the level of proline content (Fig. 7C). Conversely, *LhWRKY44* silencing reduces the drought tolerance of lily, the relative O^2−^ content was significantly higher in LhWRKY44-silenced discs than in control discs after drought treatment. The proline content was significantly decreased in LhWRKY44-silenced discs than in control discs (Fig. 7D). These results indicated that *LhWRKY44* protected lily cells from drought stress and increased the tolerance. Further, RT-qPCR analysis suggested that overexpression of *LhWRKY44* promoted the level of stress-related genes under drought treatment (Fig. 7E). Remarkably, overexpression of *LhWRKY44* led to a significant increase in the expression of stress-related genes *LhPAL* in lily compared with the controls and silencing of *LhWRKY44* significantly reduced the expression of *LhPAL* (Fig. S7A, see online supplementary material). Further examination of the interactions of LhWRKY44 protein and *LhPAL* promoter by dual-luciferase assay was conducted. The cotransformation of SK-LhWRKY44 and reporters was increased compared with the mock control, and we further proved that LhWRKY44 could bind to the W-box in fragments of *LhPAL* promoter in the EMSA assay (Fig. S7B and C, see online supplementary material). Therefore, LhWRKY44 activated stress-related genes to enhance the drought tolerance of lily, and promoted anthocyanin accumulation.

## Discussion

### LhWRKY44 positively regulates lily anthocyanin synthesis and transport

Lily is a horticultural crop with high commercial value and diverse anthocyanin pigmentation patterns [31]. As a group within the flavonoid family of plant secondary metabolites, anthocyanins determine the color of flowers and organs and protect plants from reactive oxygen species produced under abiotic stress [36, 38]. Recently, WRKY TFs are recognized as crucial regulators of anthocyanin accumulation and can function as activators or repressors based on their transcriptional activities. The WRKY proteins AtTTG2 and PhPH3 regulate flavonoid accumulation in *Arabidopsis* and *Petunia* [26, 28]. In apple, *MdWRKY11* promotes anthocyanin accumulation by enhancing the transcriptional activation of *MdMYB10* and *MdMYB11* [23]. The wounding-responsive protein MdWRKY40 is involved in apple peel anthocyanin biosynthesis [24]. In pear, PyWRKY26 binds to the *PyMYB114* promoter and increases the anthocyanin content [25]. AtWRKY75 inhibits anthocyanin biosynthesis in *Arabidopsis* [22]. BnWRKY41–1 from *B. napus* negatively regulates anthocyanin accumulation in *Arabidopsis* and inhibits the expression of *AtMYB75*, *AtMYB111*, and *AtMYBD* [39]. Similar to the function of BnWRKY41–1, MdWRKY41 negatively regulates anthocyanin and PA accumulation in apple [40]. Here, we found an anthocyanin biosynthesis-related gene, *LhWRKY44.* The transcript abundance of *LhWRKY44* showed a close correlation with the anthocyanin content (Fig. 1A and B; Fig. S1, see online supplementary material). The overexpression of *LhWRKY44* promoted anthocyanin accumulation, whereas the silencing of *LhWRKY44* decreased anthocyanin accumulation (Fig. 2). Concurrently, we showed that LhWRKY44 possesses transcriptional activity according to the dual-luciferase reporter (DLR) assay (Fig. 1E). Thus, LhWRKY44 promotes anthocyanin accumulation by acting as a positive regulator.

Anthocyanin accumulation is a complex biological process that involves synthesis by structural genes encoding enzymes and related regulatory genes in the cytoplasm and transport into vacuoles for storage with transport protein assistance after conversion to stable anthocyanins [41, 42]. The synthesis involves multiple enzyme genes, such as *PAL*, *CHI*, *CHS*, *F3H*, *DFR*, and *UFGT*. The transcriptional regulation of structural genes is tuned by regulatory genes to activate or inhibit their expression. In this study, LhWRKY44 was characterized as a positive regulator of anthocyanin accumulation in lily. Because the lily genome has not been sequenced, we identified the promoter sequences of anthocyanin-related genes using narrow homologous cloning and chromosome walking techniques according to the references [7, 12, 16]. Y1H and EMSA assay indicated that LhWRKY44 interacted with *LhF3H* promoter (Fig. 4B and C). Dual-luciferase assays further demonstrated that LhWRKY44 directly facilitated *LhF3H* promoter expression (Fig. 4A). Remarkably, in a previous study, we showed that LhGST functions as an intracellular transport protein that contributes to anthocyanin sequestration in Asiatic hybrid lilies. The silencing of *LhWRKY44* restrains the transcription level of *LhGST* (Fig. 2F). A dual-luciferase assay analysis revealed that LhWRKY44 induced the expression of *LhGST*, which suggested that LhWRKY44 might regulate *LhGST* (Fig. 4A and C). Subsequently, the results from Y1H assays and EMSAs further revealed that LhWRKY44 directly bound to the W-box sequence on the *LhGST* promoter (Fig. 6C and D). This study provides the first demonstration that LhWRKY44 participates in GST-mediated transport process.

Altogether, we generated the regulatory system of LhWRKY44 on anthocyanin accumulation in monocot lily flowers. LhWRKY44 participates in both anthocyanin synthesis and transport by binding to the *LhF3H* and *LhGST* promoters to positively regulate lily anthocyanin accumulation. Anthocyanin-related WRKY genes provide support for the functional differentiation of anthocyanin accumulation in different plants.

### LhWRKY44 and LhMYBSPLATTER harbor a dual WRKY-MBW module

Many researchers are committed to studying the formation of a quaternary complex of WRKY-MBW, including WRKY and the MYB-bHLH-WD40 ternary complex. MdWRKY40 interacts with MdMYB1 to coregulate apple wounding-induced anthocyanin accumulation [24]. PyWRKY26 functions with PybHLH3 and directly activates the *PyMYB114* promoter to increase red-fleshed pear anthocyanin accumulation [25]. In *Petunia*, the regulatory network of the PH3 TF only joined the PH4 − AN1 − AN11 (MBW) complex by interacting with the WD40 protein AN11 and activated the expression of *PH5* to regulate vacuolar acidification for anthocyanin storage [28]. Similarly, in *Arabidopsis*, TTG2 interacts with the WD40 protein TTG1 of the MBW complex to regulate the *TT12* and *TT13*/*AHA10* genes [26]. In Asiatic hybrid lilies Tango series cultivars, LhMYBSPLATTER, LhbHLH2 and LhWDR activators co-function on anthocyanin accumulation as MBWcomplex [7, 14, 16, 18]. A phylogenetic analysis showed that LhWRKY44 exhibits a close evolutionary relationship with PH3 and TTG2, which means that they may have a similar function and regulatory mechanism (Fig. 1C). Therefore, we investigated whether the same mechanism also exists in lily. BiFC and Y2H assays showed that LhWRKY44 did not interact with LhWDR protein (Fig. 3A–C). Moreover, the expression of *LhPH5* (a homolog of *AHA10*) isolated from ‘Tiny Padhye’ was not triggered by LhWRKY44 in LhWRKY44-silenced tepals (Fig. S8, see online supplementary material). However, we noted that LhWRKY44 could interact with the LhMYBSPLATTER protein and thus enhanced the effect of the LhMYBSPLATTER-LhbHLH2 activator on anthocyanin accumulation (Fig. 3F and G).

In addition, LhWRKY44 could also activate *LhMYBSPLATTER* expression and bound to the W-box motif of the *LhMYBSPLATTER* promoter (Fig. S5, see online supplementary material). These results indicated that the LhWRKY44-LhMYBSPLATTER module played a pivotal regulatory role in anthocyanin accumulation that differs from that of PH3 and TTG2, which participated in vacuolar acidification. The WRKY/WD40 regulatory module may have diverged since the evolutionary separation of these plant species. Hierarchical mechanisms of LhWRKY44 and LhMYBSPLATTER, a component of the MBW activation complex, may be an important feature of anthocyanin regulation in lily tepals.

### LhWRKY44 may be a major regulator that integrates the light and drought-induced anthocyanin biosynthesis

The accumulation of anthocyanins in plant vegetative tissues and flowers is affected by internal factors and external environmental factors such as light, temperature, hormones, sugar, etc. [24, 43]. WRKY TFs participate in light signaling pathways. In *Arabidopsis*, AtWRKY40 and AtWRKY63 activate the transcription of light signaling pathway genes [44]. In apple, light induces *MdWRKY1* expression and promotes anthocyanin accumulation [20]. *Cis*-element analysis of the *LhWRKY44* promoter revealed a large number of light-responsive elements, such as GT1-motif, Sp1, MRE, LAMP-element, Gap-box, and TCT-motif (Table S1, see online supplementary material). The light treatment assay of lily flowers proved that LhWRKY44 is a component of light signaling. Light promoted anthocyanin accumulation in lily tepals and induced the expression of *LhWRKY44* (Fig. 5A–C). This result is similar to that for results of *PpWRKY44* in pear [30].

Additionally, further results showed the expression of *LhWRKY44* was also induced by NaCl, ABA and 4°C treatment, especially drought treatment (Fig. 5D). Here, we proved that drought condition promoted anthocyanin biosynthesis in lily and *LhWRKY44* remarkably improved drought resistance (Figs 6 and7). *LhWRKY44* may regulate anthocyanin biosynthesis by responding to drought stress. Phenylalanine ammonia-lyase (PAL) is a key enzyme in the phenylpropanoid metabolism pathway, and is also an initial and upstream enzyme of anthocyanin biosynthesis pathway [45]. Here, we proved that *LhWRKY44* bound to *F3H* and *GST*, and activated their expression as a downstream factor of the anthocyanidin synthesis pathway. Researches indicated that PAL enhanced the resistance of biotic and abiotic stress by synthesizing secondary substances, such as lignin, phytoalexin, and phenol. The change of PAL activity is often a significant physiological indicator of plant stress resistance [46]. Dual-luciferase assay and EMSA indicated LhWRKY44 activated and bound to the promoter of *PAL* (Fig. 7F and G). Thus, we considered that *LhPAL* acts as a stress-related gene to jointly function with LhWRKY44 in drought response signals. Overall, LhWRKY44 enhanced the drought tolerance by activating stress-related gene expression to promote anthocyain accumulation in lily. Whereas if there any other TFs to co-regulated anthocyanin biosynthesis with LhWRKY44 when integrating the light and drought response signals. The precise examination mediated by LhWRKY44 of anthocyanin pigment remains to be further completed.

### LhWRKY44 may be involved in development signal pathway

Anthocyanin performs a protective role by providing a light-absorbing screen for photosynthetic cells and scavenging reactive oxygen during stress conditions. In vegetative tissues of plants (i.e., leaves and flowers), there must be stringent regulatory mechanisms that respond to both environmental and developmental cues to fulfill the function of anthocyanins [47]. Research conducted over the past few decades has highlighted the critical roles of WRKY TFs in plant stress, flowering, development and other processes [48]. During the stress assays, we accidentally noted that the overexpression lines promoted flowering and significantly up-regulated the expression of genes related to flowering, such as *AtAP1*, *AtFT*, and *AtSOC1* (Fig. S9, see online supplementary material). Although results need to be further confirmed in lily, it seems to explain the confusing question of what specifically determines the spatial expression of *LhWRKY44* in lily tepals. LhWRKY44 is associated with light-induced and drought stress-induced anthocyanin accumulation, whereas the level of *LhWRKY44* increased with deepening of the basal tepal color compared with that in the upper areas in the Tango series cultivars when these tepals were under the same conditions (Figs 1A and B and5A–C). Here, we conjecture that the internal development signal may specifically determine the expression of *LhWRKY44* spatially. However, the specific mechanism of LhWRKY44-mediated environmental and development signal pathways to determine the timing, level, and patterning of anthocyanin accumulation remains unclear and will be the focus of future research. Our findings may provide insights into the precise gene activation regulation of anthocyanins during development or in response to environmental cues, whereas more comprehensive studies on whether interactions between the genetic developmental factors and external environmental factors occur during the development of lily for joint regulation of anthocyanin accumulation are needed. Our findings may provide insights into the precise gene activation regulation of anthocyanins during development or in response to environmental cues.

## Materials and methods

### Plant materials, growth conditions, and treatments

Lily cultivars were grown in a greenhouse located at the Institute of Vegetables and Flowers, Chinese Academy of Agricultural Sciences (Beijing, China). Flower samples were collected at five stages defined according to a previous method with minor modifications [15]. *Arabidopsis thaliana* (Col-0) and tobacco plants (*N. tabacum* and *N. benthamiana*) were grown in nutritive soil with 70% relative humidity in a programmable incubator with a 16-h photoperiod at 23°C. Calli of ‘Orin’ apple were subcultured in Murashig and Skoog medium (MS) supplemented with 1.5 mg^**.**^L^−1^ 2,4-dichlorophenoxyacetic acid (2,4-D) and 0.5 mg^**.**^L^−1^ 6-benzylaminopurine(6-BA) at 23°C in continuous darkness at 15-day intervals.

The method previously used to determine the effect of light on anthocyanins in lily with a modification [49] was used. ‘Tiny Padhye’ with 1–2-cm-long flower buds were grown in dark and light (natural light supplemented with white fluorescent bulbs using LEDs; PPFD: 135.29 ± 1.46–173.98 ± 1.20 mmol^−2^ s^−1^) conditions for 12 d in an incubator at 23°C. All samples were immediately chopped, frozen and stored at −80°C.

### Stress tolerance analysis

One-month-old seedlings (budding stage) were used to determine the expression *LhWRKY44* under different stress treatments. We collected the samples at 0, 12, 24, and 36 h after treatment with 100 μmol/L ABA, PEG6000 (30%), 200 mmol/L NaCl, and 4°C. For drought stress tolerance assay, the Arabidopsis seedlings were cultured in 1/2MS medium with mannitol (0 mM,100 mM, and 200 mM). After 5 days, the main roots were photographed and measured. All seedlings were transplanted into soil and conducted 20 days of drought treatment, the survival rates were calculated. PEG 6000 (10%) was applied to lily tepal discs. After 72 h, the samples were collected for further analysis.

### Total RNA extract and RT–qPCR analysis

Total RNA was extracted using an RNA Prep Pure Plant Kit (TIANGEN, Beijing, China). cDNA was synthesized by TransScript® II One-Step gDNA Removal and cDNA Accumulation SuperMix (TransGen Biotech, Beijing, China). RT–qPCR was performed by Hieff® qPCR SYBR® Green Master Mix (YEASEN, Shanghai, China). The relative expression levels were calculated with the 2^−ΔΔCt^ method. The qPCR primers were listed in Table S2 (see online supplementary material).

### Anthocyanin, O^2−^ and proline content measurement

Total anthocyanin extraction and measurement were performed as described previously [7]. O^2−^ and proline content were measured by the reagent kits (Solarbio Science & Technology, Beijing, China). At least three biological replicates of each sample were included.

### RACE assay and bioinformatics analysis

Full-length LhWRKY44 was amplified by the GeneRacer kit (Invitrogen, Carlsbad, CA, USA) using the primers in Table S3 (see online supplementary material). Multiple sequence alignments were conducted by DNAMAN with the default parameters. Using MEGA, phylogenetic trees were constructed with the neighbor-joining (NJ) algorithm method and 1000 bootstrap replicates.

### Transcriptional activity analysis and subcellular localization

Vector construction of transactivation activity assays of LhWRKY44 in tobacco leaves were performed following a previous method [50, 51]. To generate a subcellular localization fusion protein, the CDS of LhWRKY44 was inserted into a pCAMBIA2300-eGFP vector. All recombinants were transformed into *Agrobacterium tumefaciens* strain GV3101 and were infiltrated into *N. benthamiana* leaves. After 3 d, the ratios of LUC to REN were measured by the Dual Luciferase Reporter Gene Assay Kit (Promega, Madison, WI, USA) with a luminometer (Promega). The activity was analysed with at least three biological replications. LUC signals in living images were captured by a CCD camera. The GFP signals in leaf epidermal cells were imaged after 3 d of infiltration into tobacco leaves by confocal laser microscopy.

### Stable genetic transformation of apple calli and Arabidopsis

LhWRKY44-OX apple calli were generated and screened by genetic transformation following a previous report [20]. After obtaining a sufficient number of transgenic calli, the calli were stored in a phytotron for anthocyanin accumulation. *A. thaliana* was transformed according the floraldip method [7]. Transgenic lines were selected on medium with 10% Basta. All primers are listed in Table S4 (see online supplementary material).

### Transient overexpression and silencing of *LhWRKY44* gene in lily

To confirm the function of LhWRKY44 on anthocyanin accumulation in lily, we transiently silenced *LhWRKY44* in lily cultivar ‘Tiny Padhye’ basal tepals according to a previously described method [33]. Briefly, for the generation of pTRV2-LhWRKY44, specific fragments of ~240 bp of LhWRKY44 were amplified and infused into the pTRV2 vector. Agrobacterium EHA105 cultures containing combinations of pTRV1, pTRV2, or its derivatives were mixed at a 1:1 ratio before infiltration and were then introduced into the outer tepals of the colored buds close to stage 3 (approximately 4–5 cm). At least 10 plants per replicate of each treatment and three biological replicates were included in the experiment. Digital photographs were taken after 1 week. Similarly, the transient expression assay in lily leaves and scales were performed by vacuum infiltration as previously [34, 35] described with minor modifications. Water and empty infiltrations were used as negative controls. Leaves and scales of the same part were washed on the surface with tap water and uniformly cut into 1.5 cm segments, and intermediate scales were cut into discs with a diameter of approximately 1 cm by a puncher for vacuum infiltration. Each treatment consisted of at least 20 leaf and 60 scale discs per replicate, with three experimental replicates. The transient overexpression and TRV-VIGS of lily tepal discs in drought tolerance assay was performed according to the procedures described [37]. The upper lily tepals of the colored buds close to stage 4 were conducted.

### Dual-luciferase assay

DLR assays were performed as previously described [34]: a pGreenII 0029 62-SK vector was fused to the CDS of LhWRKY44 as the effector and with a pGreenII 0800-LUC vector carrying the promoter sequences as reporters. The primer sequences used in the DLR assay are shown in Table S4 (see online supplementary material). The pGreenII 0029 62-SK empty vector was used as a control. Subsequently, the GV3101 bacterial solution containing the reporter and effector constructs was transiently introduced into tobacco leaves. The luciferase signals were imaged and measured as mentioned above.

### Y1H assay

Y1H assays were performed using the Matchmaker Gold Yeast One-Hybrid Library Screening System (Clontech). The sequence of LhWRKY44 fused to the pGADT7 vector as prey. The promoters fused to the pAbAi vector as bait and then transformed into the Y1H Gold yeast strain. The transformants were spread on SD/-Ura/AbA culture medium for autoactivation tests. The primers are listed in Table S4 (see online supplementary material). Subsequently, the physical interaction was determined according to colony growth on SD/−Leu medium supplemented with corresponding AbA concentrations, and the colonies were to grow for 3–5 d at 28°C.

### EMSA

To obtain soluble protein, LhWRKY44 was cloned into the pET-32a vector (with 6× His-tag) to produce recombinant LhWRKY44 protein with a polyhistidine (His) tag in *Escherichia coli* strain BL21 (DE3). Ni-NTA Sefinose™ Resin (Sangon, Shanghai, China) was subsequently used to purify the proteins. EMSA was performed following the LightShift™ Chemiluminescent EMSA Kit (Thermo Fisher Scientific, Waltham, MA, USA). The specific primer sequences used in the EMSAs are listed in Table S4 (see online supplementary material).

### BiFC assay

The LhWRKY44 and LhMYBSPLATTER CDSs lacking stop codons were inserted into the P2YC and P2YN vectors, respectively. The primers are presented in Table S4 (see online supplementary material). The GV3101 bacterial solution containing the resultant vectors with the nuclear marker were introduced into *N. benthamiana* leaves as described previously [52] and visualized by confocal laser microscopy after 3 d of transformation.

### Y2H assay

The sequence of LhWRKY44 fused to pGADT7 as a prey vector. The sequences of LhMYBSPLATTER, LhbHLH2, and LhWDR were individually amplified into pGBKT7 to yield bait vectors. The prey and baits were cotransformed into *Saccharomyces cerevisiae* strain AH109. The assay was performed according to the Matchmaker GAL4 Two-hybrid System (Clontech). The interactions were detected on DDO (SD medium lacking Leu and Trp) and QDO/X (SD medium lacking Leu, Trp, His and Ade, with X-α-gal) media.

### FLC assay

The vector information and protocol for the FLC assay were based on previous studies [53]. Additionally, LhWRKY44 was incorporated into the pGreenII 0029 62-SK vector for subsequent overexpression. The recombinant plasmids were transformed into GV3101, and different combinations were then used for the infiltration of tobacco leaves as mentioned above. The LUC signal was imaged by a CCD imaging camera.

### Co-IP assay

The Co-IP assay was conducted according to a previously described method [54]. Full-length LhWRKY44 and LhMYBSPALTTER were obtained by PCR amplification and fused to the N-terminus of FLAG tags and YFP-HA, respectively. The Agrobacterium strain GV3101 harboring LhWRKY44-FLAG was injected into tobacco leaves together with YFP-HA and LhMYBSPALTTER-YFP-HA recombinant vectors. After 2 d, we collected equal numbers of samples to extract total protein in extraction buffer and incubated them with anti-HA antibody conjugated beads. Immunoblots of the input and the IP product were conducted with an anti-FLAG antibody, and the bands were imaged using a chemiluminescent imaging system.

### Statistical analysis

The asterisks indicate significant differences from three biological replicate experiments by a *t* test (^*^P < 0.05, ^**^P < 0.01, and ^***^P < 0.001). Different letters indicate significant differences (*P* < 0.05) revealed by ANOVA followed by Tukey’s correction.

## Acknowledgments

The authors thank the editor and the anonymous reviewers for their efforts to improve the manuscript. This study was supported by the National Natural Science Foundation of China (32172624, 32172612, 31672196) and the Programs for National Key R & D Plan (2019YFD1001002). We thank Prof. Nan Ma, Prof. Xia Cui, Prof. Allen A C, and Prof. Yuanwen Teng for the providing experimental plasmids and guidance, and we thank Prof. Ji Tian for providing apple calli materials.

## Author contributions

J.M. and M.B. conceived and designed the experiments. M.B. and J.M. wrote the paper. M.B. performed the experiments. R.L., J.W., Y.Q., X.L., and Y.C. provided assistance. G.H., Y.Y., P.Y., and L.X. helped to revise the article. All authors read and approved the manuscript for submission.

## Data availability

All relevant data in this study are provided in the article and its supplementary data files.

The gene accession numbers used can be found in National Center for Biotechnology Information (NCBI) database, and they are listed in (Table S5, see online supplementary material).

## Conflict of interest statement

The authors declare no competing interests.

## Supplementary data

Supplementary data is available at *Horticulture Research* online.

## Supplementary Material

Web_Material_uhad167
