# Supplementary material for: Multifaceted roles of LhWRKY44 in promoting anthocyanin accumulation in Asiatic hybrid lilies (Lilium spp.)
Source: Hortic Res. 2023 Aug 22;10(9):uhad167. doi: 10.1093/hr/uhad167 (PMC10535013; doi:10.1093/hr/uhad167)
Supplement: Web_Material_uhad167 [file web_material_uhad167.zip › clean version-Supporting Figures.docx]

**Supporting Figures**


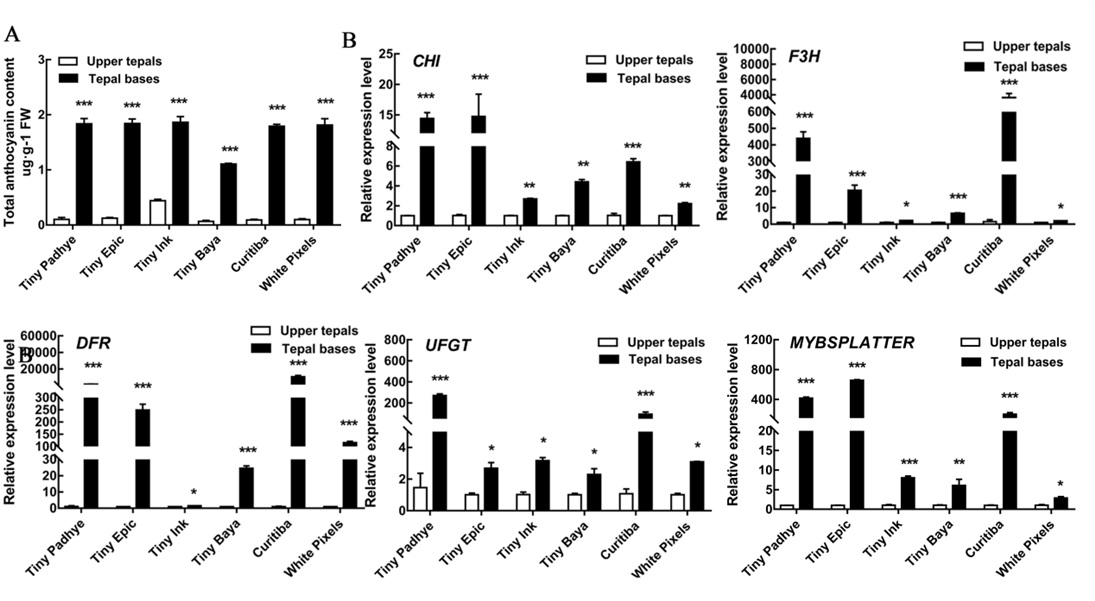


**Figure S1 Expression patterns of anthocyanin biosynthesis-related genes in the Tango series lily cultivars*.*** (A) Determination of the total anthocyanin content of Tango series lily cultivars.‘Tiny Padhye’;‘Tiny Epic’; ‘Tiny Ink’;‘Tiny Baya’; ‘Curitiba’;‘White Pixels’. (B) Relative transcript levels of anthocyanin biosynthesis-related genes. Mean values ± SDs are shown at least three biological replicates. Asterisks represent T-test statistically significant differences (*, P < 0.05; **, P < 0.01; ***, P < 0.001.


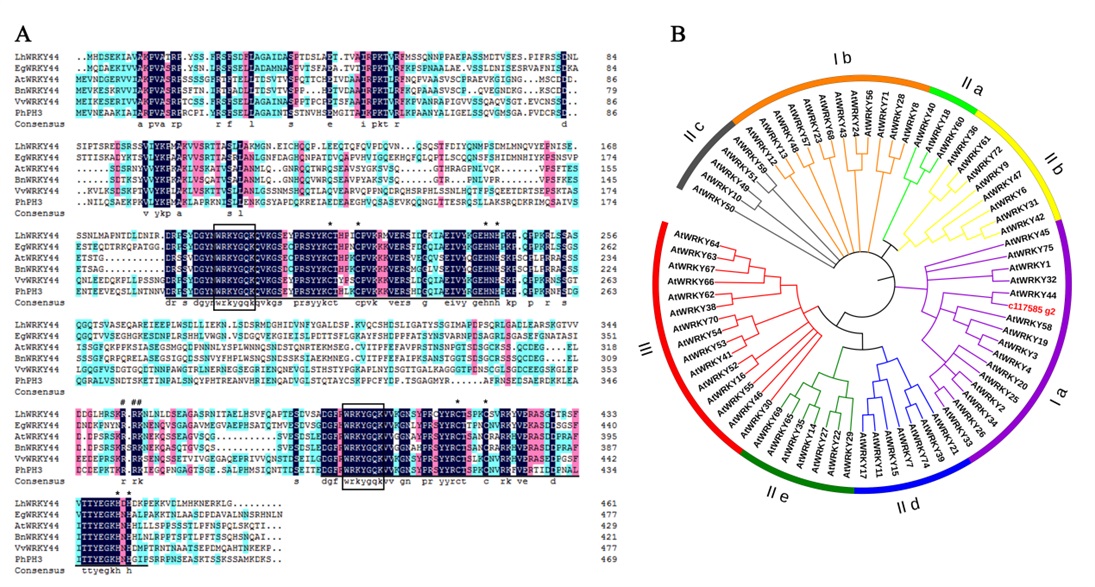


**Figure S2 Multiple sequence alignment and phylogenetic tree analysis of LhWRKY44.** (A) Multiple amino acid sequences alignment of WRKY44 from different plants. Lh: *Lilium hybrid*(LhWRKY44, OP380462), Eg: *Elaeis guineensis* (EgWRKY44, XP_010908965), At: *Arabidopsis thaliana* (AtWRKY44, NP_181263), Bn:*Brassica napu*s (BnTTG2, XP_013716377), Vv: *Vitis vinifera* (VvWRKY44, XP_002275978), Ph:*Petunia hybrida* (PhPH3, AMR43368). The 60 amino acids of the WRKY domain is marked by a black underline, and the highly conserved domain WRKYGQK is boxed. The nuclear localization signal, KRRK, is marked by pound signs (#), and the C_2_H_2_ zinc finger is marked by asterisks (*). (B) Phylogenetic tree analysis of LhWRKY44 with other WRKY proteins from *A. thaliana.* LhWRKY44 (c117585_g2) is highlighted. Accession numbers: Partial sequences of WRKY family genes in *A. thaliana* were downloaded from Arabidopsis database (<http://www.arabidopsis.org/index.jsp>), the accession numbers can be found in the database.

**
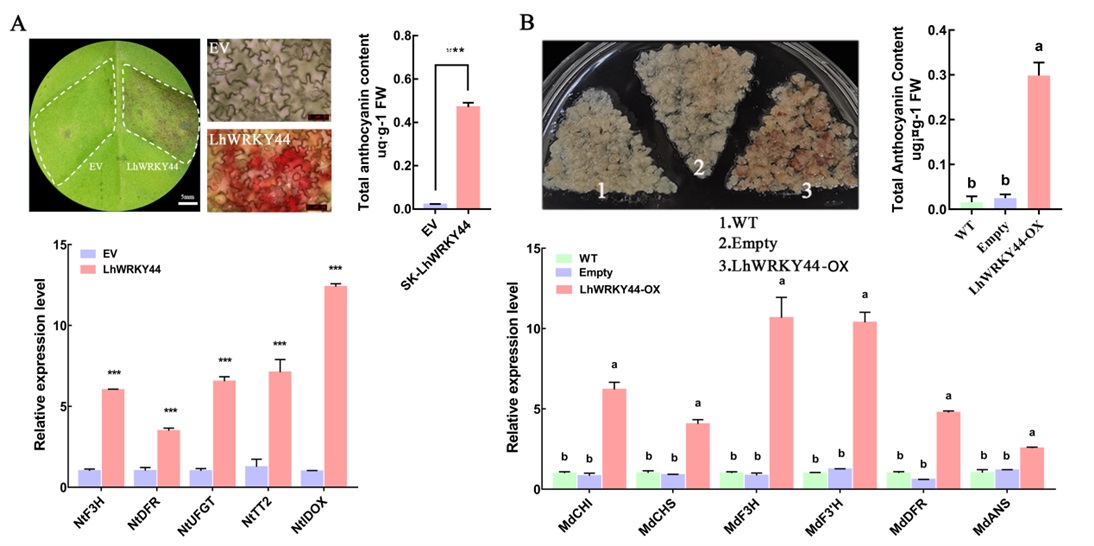
**

**Figure S3 Heterologous overexpression *LhWRKY44* gene in tobacco leaves and apple calli.** (A) Production of anthocyanin by transient overexpression of *LhWRKY44* (SK-LhWRKY44) and EV(SK empty vector) in *N. tabacum* leaves. The surfaces of leaves were photographed by camera and stereomicroscope. Bar, 5mm. (B) The heterologous stable overexpression of *LhWRKY44* gene facilitated anthocyanin accumulation in apple calli. WT, wild-type ‘Orin’ apple calli; Empty, empty vector, negative control; LhWRKY44-OX, *LhWRKY44*-overexpressing apple calli. Representative pictures were shown.


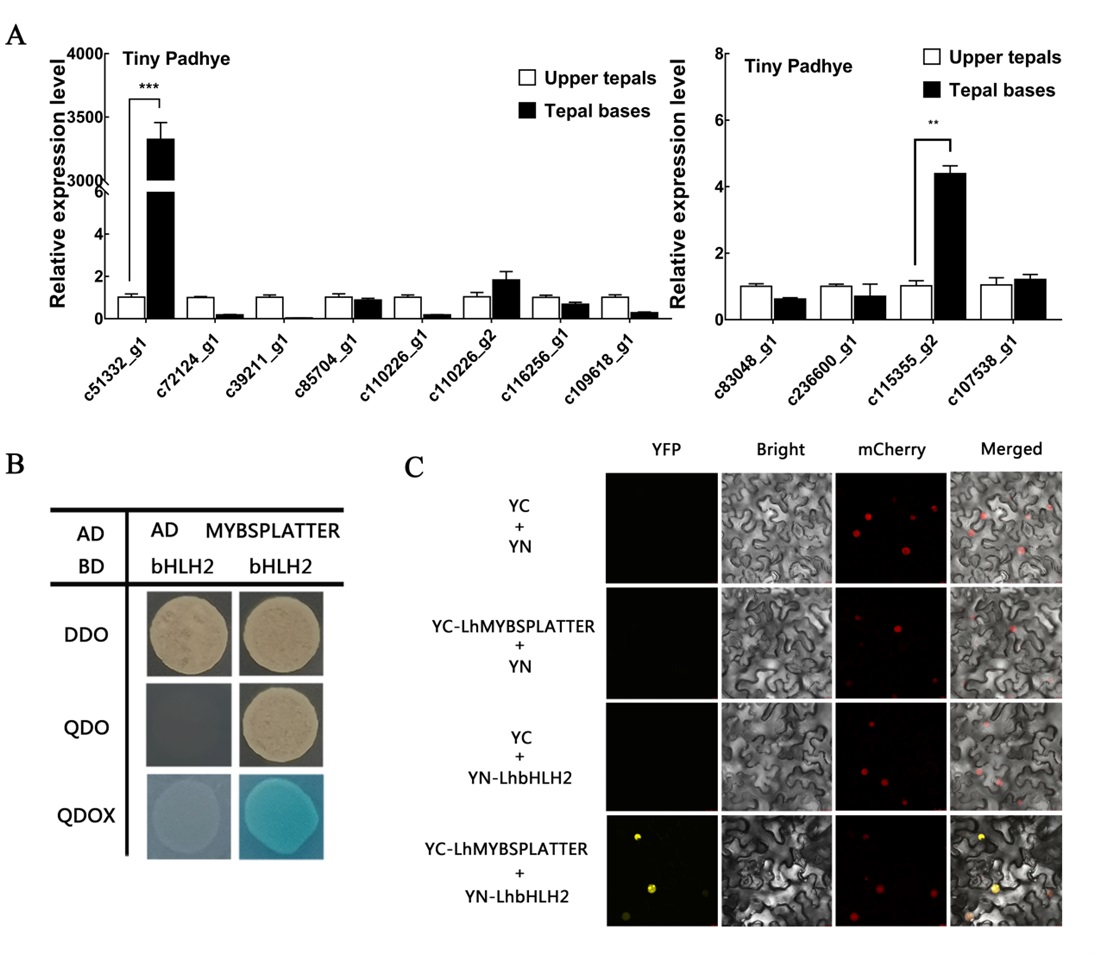


**Figure S4 LhMYBSPLATTER and LhbHLH2 form MBW complex and play roles in anthocyanin biosynthesis.** (A) Expression of MYB TFs belonging to SupGroup5 and SupGroup6, and bHLH TFs belonging to SupGroupIIIf in transcriptome data. Flowers (Stage 3) were subjected to RNA extraction. The data represent the mean ± SD of at least three biological replicates. Asterisks represent T-test statistically significant differences (**, P < 0.01; ***, P < 0.001). Y2H (B) and BiFC (C) assays of LhMYBSPLATTER(c51332_ g1) and LhbHLH2(c115355_ g2).

**
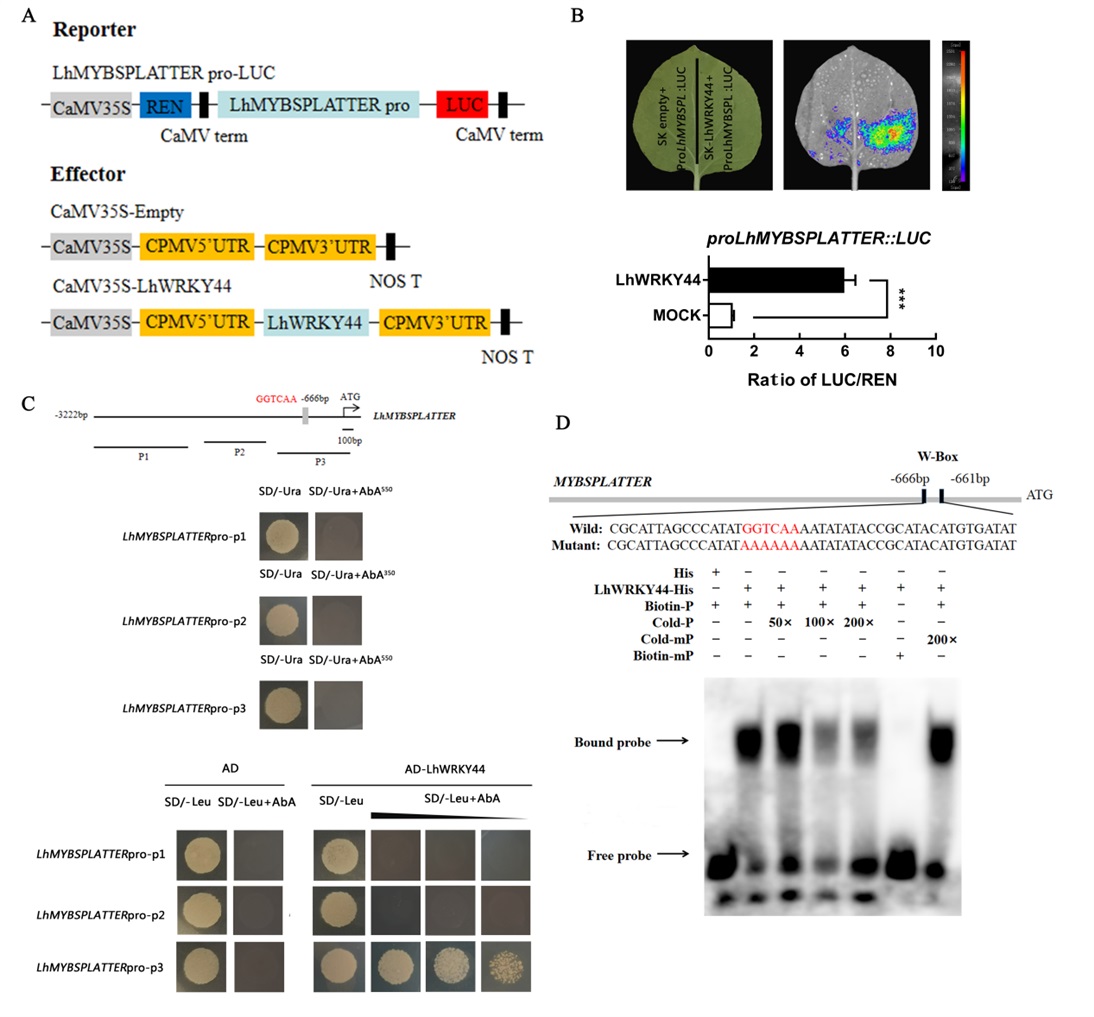
**

**Figure S5 LhWRKY44 activates *LhMYBSPLATTER* and binds W-box sequence on the *LhMYBSPLATTER* promoter.**

(A)Dual-luciferase assay of LhWRKY44 and *LhMYBSPLATTER* promoter. (B) Relative luciferase activity and fold changes. Asterisks represent T-test statistically significant differences (***, P < 0.001) , error bar, SD. (C) Y1H assay showing prey pGADT7-LhWRKY44 interacts with bait pAbAi-*LhMYBSPLATTERpro*-p3. (D)EMSA showing that LhWRKY44 binds to the probe containing W-box sequence on *LhMYBSPLATTER* promoter.

**
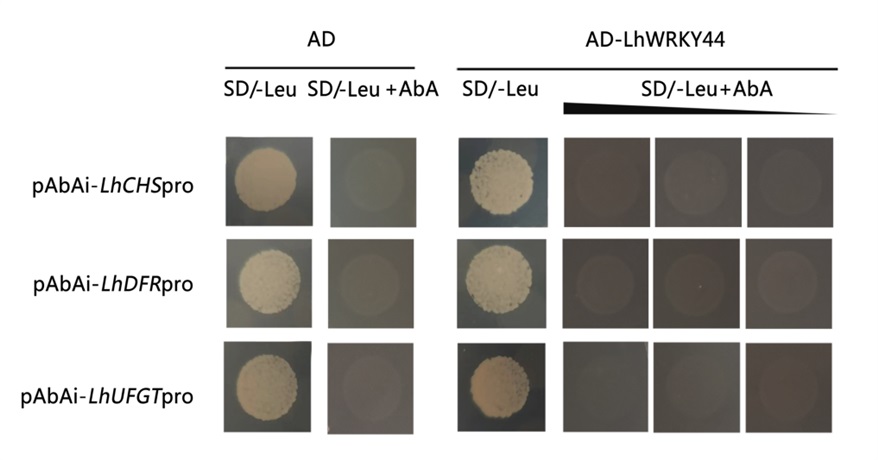
**

**Figure S6** LhWRKY44 not directly binds to *LhCHS, LhDFR and LhUFGT* promoters. Yeast one-hybrid analysis of the interaction of LhWRKY44 and anthocyanin biosynthetic gene promoters.


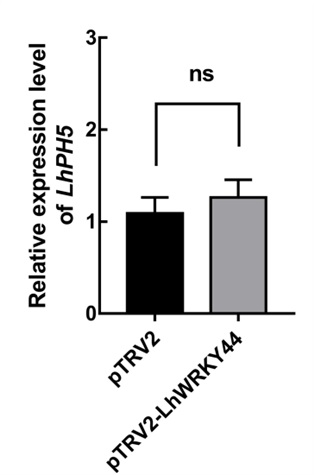


**Figure S7 Expression analysis of *LhPH5.*** Expression level of *LhPH5* after silencing *LhWRKY44* in lily tepals. The data represent the mean ± SD of at least three biological replicates. ns represent no significant differences, as determined by T test.

**
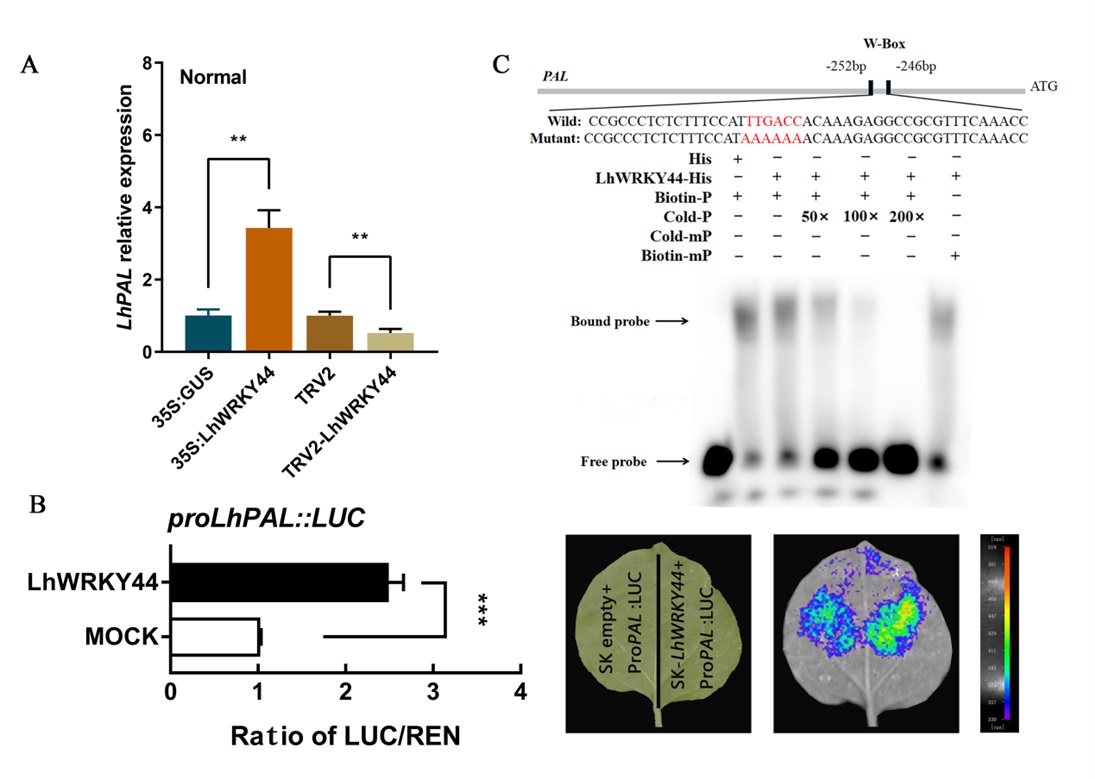
**

**Figure S8** LhWRKY44 enhanced drought tolerance by interacting with stress-related gene *LhPAL*. (A) The expression of stress-related genes *LhPAL* in treated lily tepal discs. (B) *LhWRKY44* activated *LhPAL* promoter expression in dual-luciferase assay. (C) LhWRKY44 binds to the W-box element of the *LhPAL* promoter in an EMSA. The asterisks indicate significant differences identified by a T-test (*P < 0.05, **P < 0.01 and ***P < 0.001); error bar, SD.

**
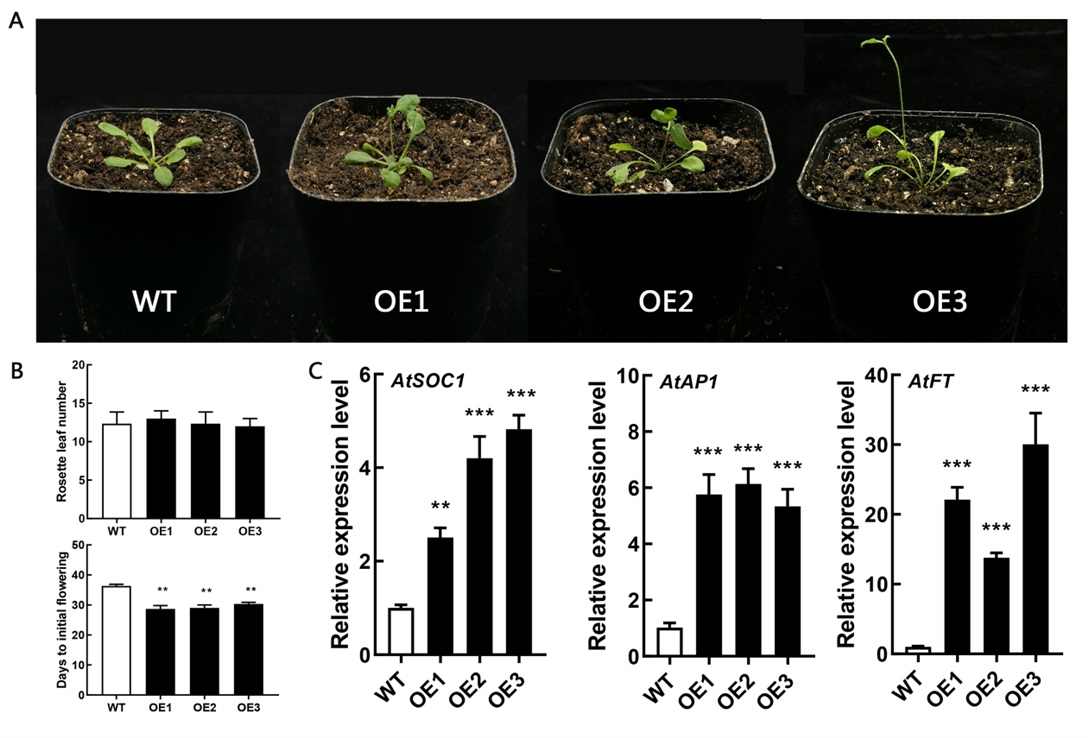
**

**Figure S9 Overexpression of *LhWRKY44* confers early flowering in *Arabidopsis*.** (A) Phenotypic analysis of transgenic Arabidopsis overexpressing *LhWRKY44*. (B) Number of rosette leaves of *LhWRKY44* transgenic lines and WT plants. (C) Days to initial flowering of *LhWRKY44* transgenic lines and WT plants. (D) Expression analysis of flowering-related genes in transgenic Arabidopsis and WT plants. The data represent the mean ± SD of at least three biological replicates. The asterisks indicate significant differences identified by a T-test (*P < 0.05, **P < 0.01 and ***P < 0.001).
