# Supplementary figures and images for: Multifaceted roles of LhWRKY44 in promoting anthocyanin accumulation in Asiatic hybrid lilies (Lilium spp.)
Source: Hortic Res. 2023 Aug 22;10(9):uhad167. doi: 10.1093/hr/uhad167 (PMC10535013; doi:10.1093/hr/uhad167)

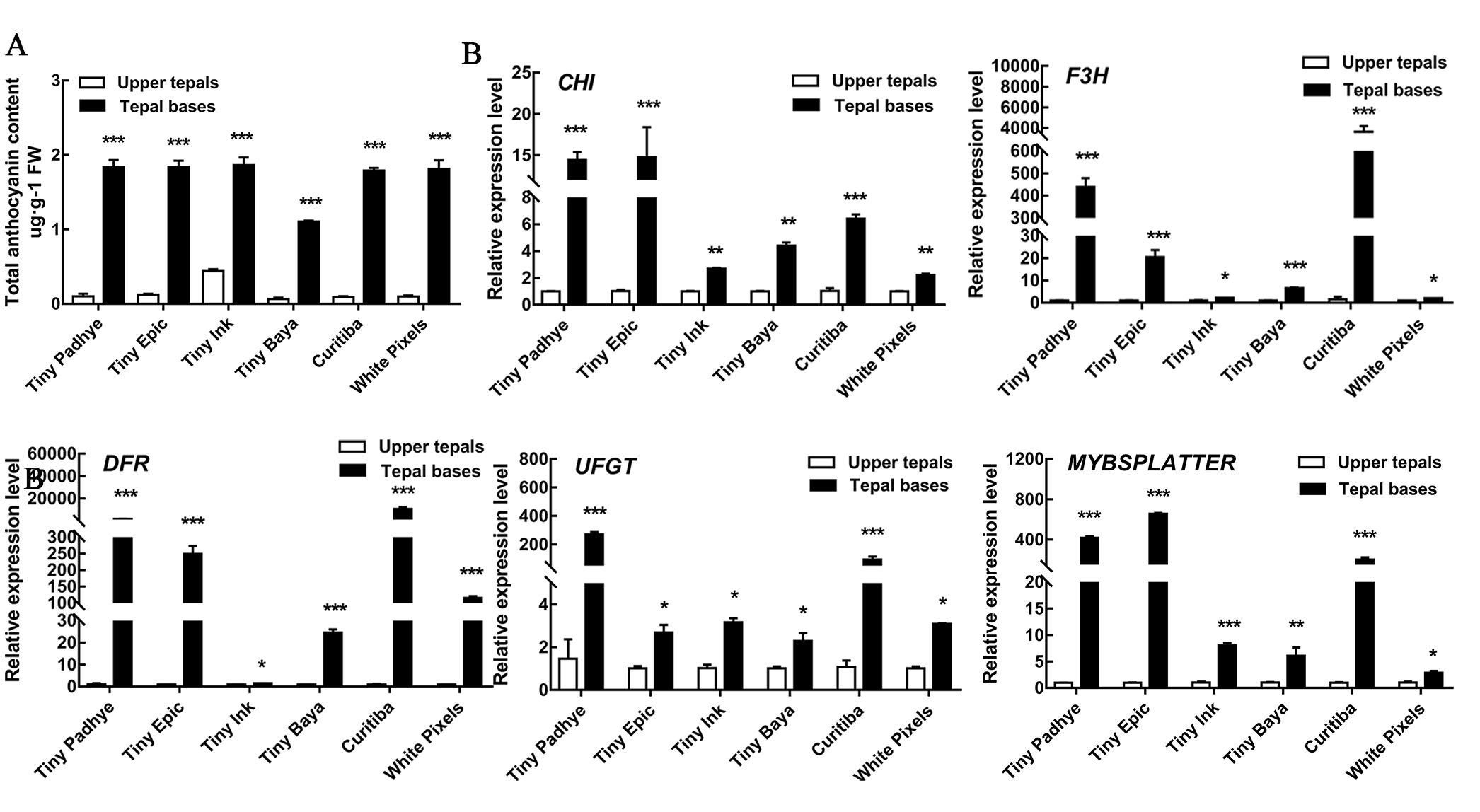

Supplement: Web_Material_uhad167 [file web_material_uhad167.zip › Figure S1.tif]

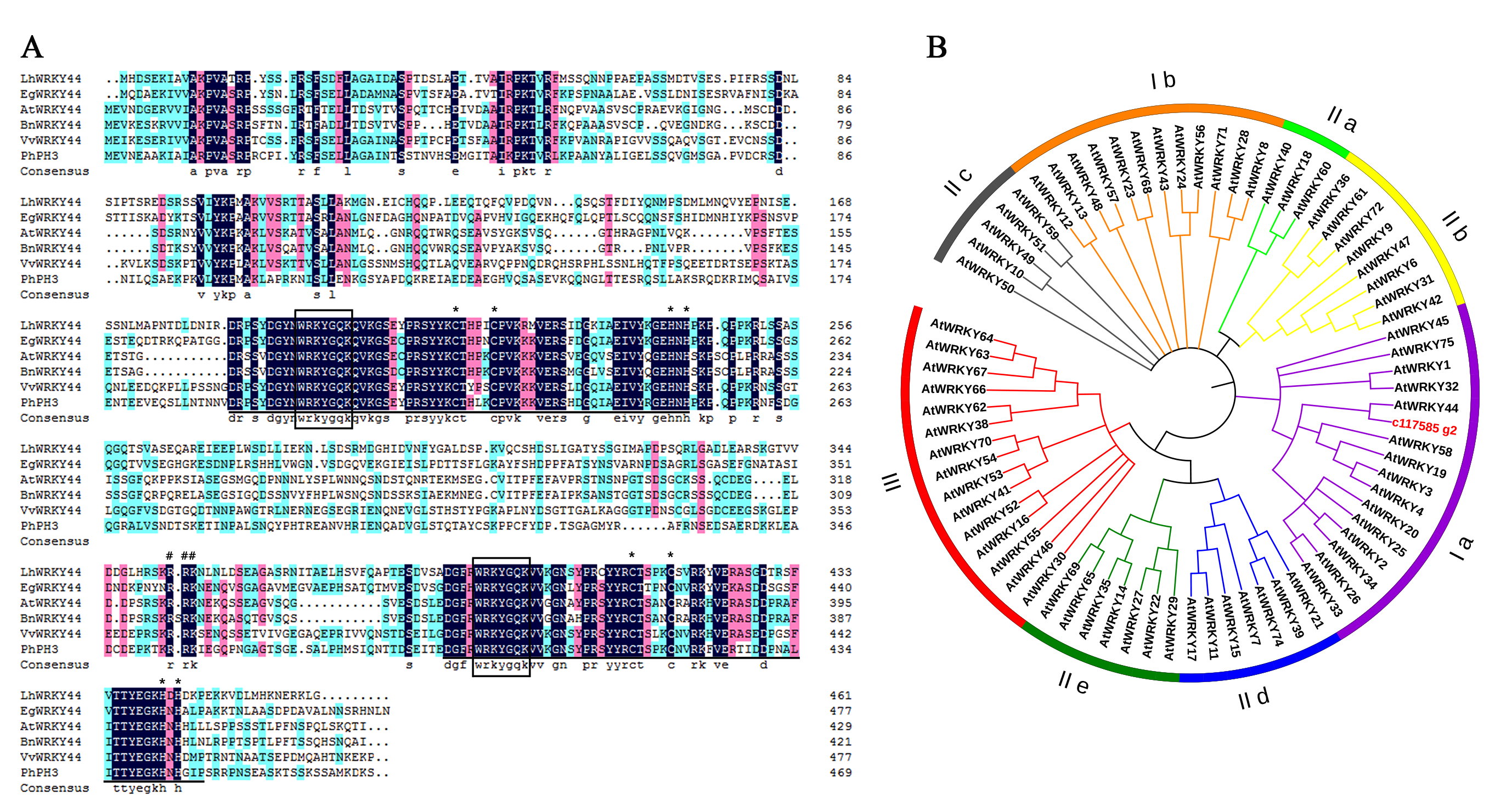

Supplement: Web_Material_uhad167 [file web_material_uhad167.zip › Figure S2.tif]

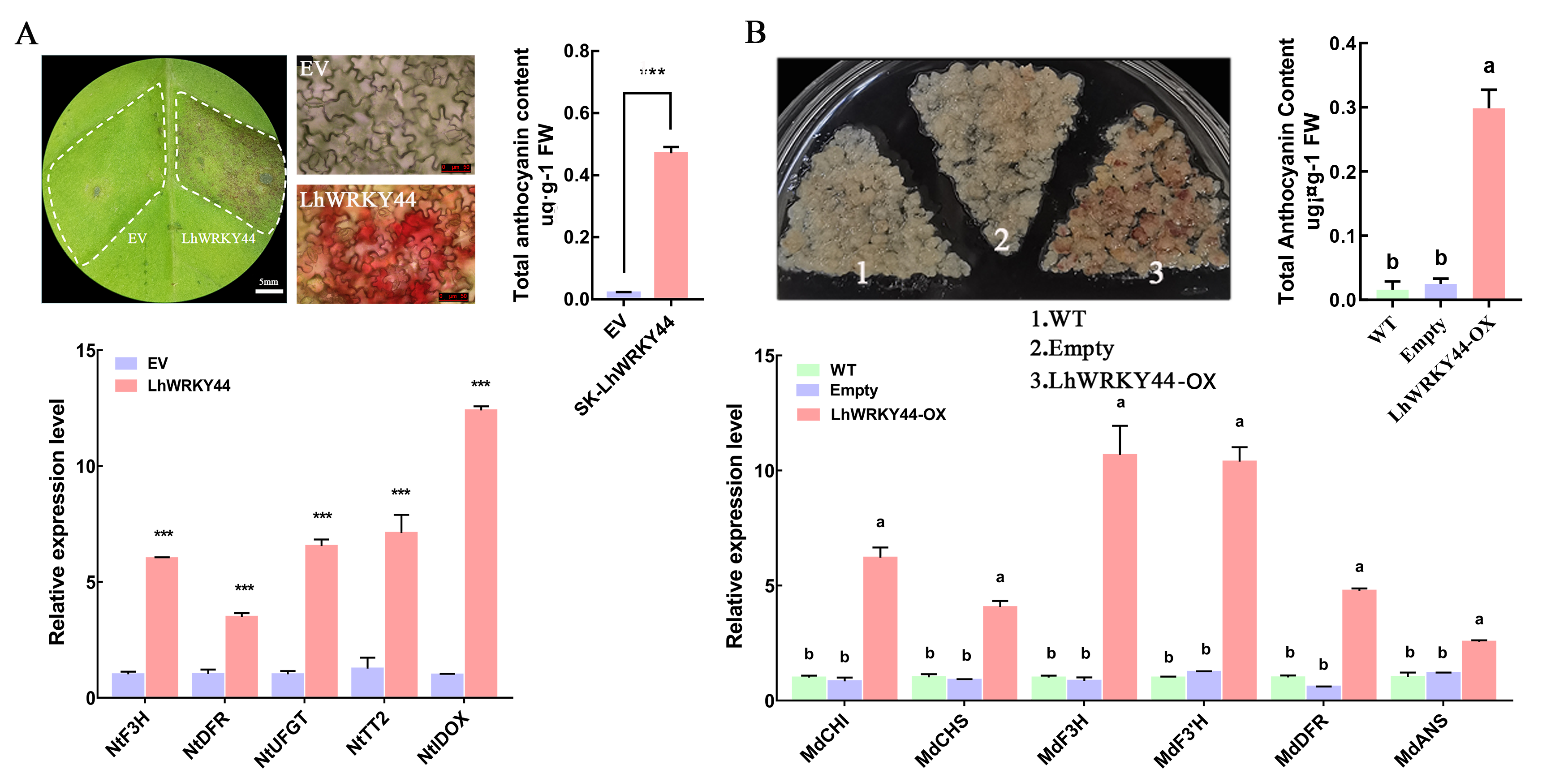

Supplement: Web_Material_uhad167 [file web_material_uhad167.zip › Figure S3.tif]

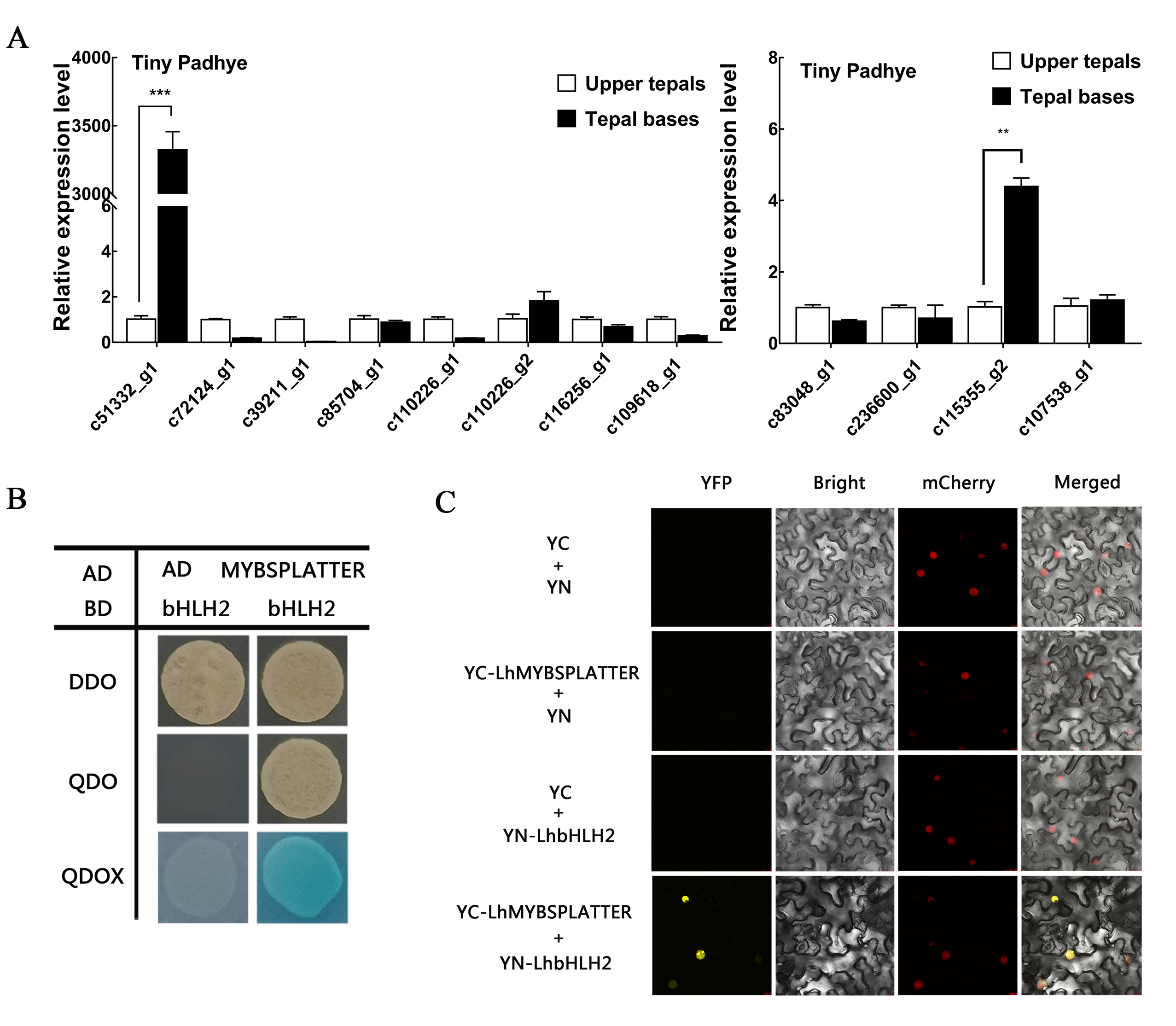

Supplement: Web_Material_uhad167 [file web_material_uhad167.zip › Figure S4.tif]

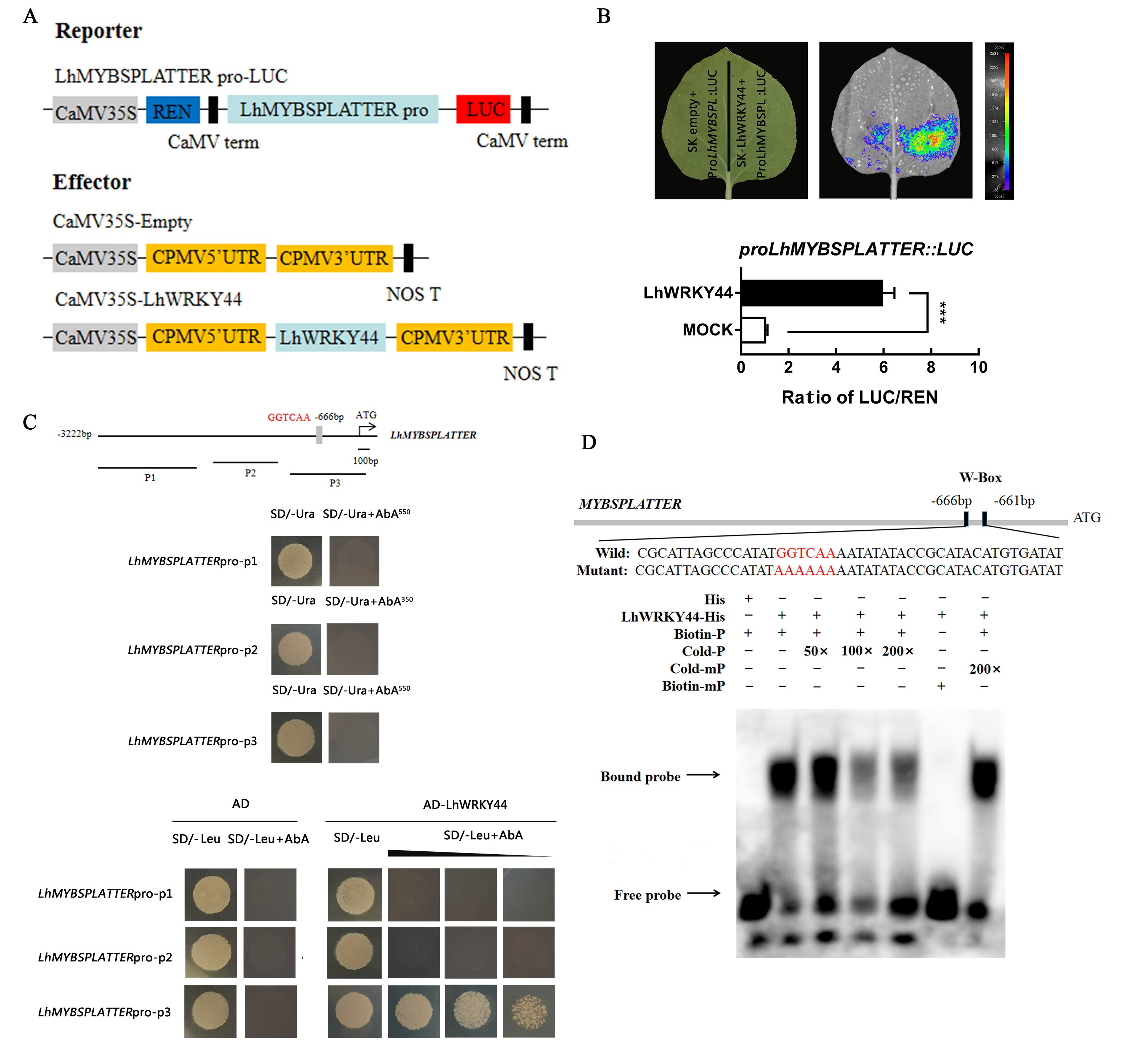

Supplement: Web_Material_uhad167 [file web_material_uhad167.zip › Figure S5.tif]

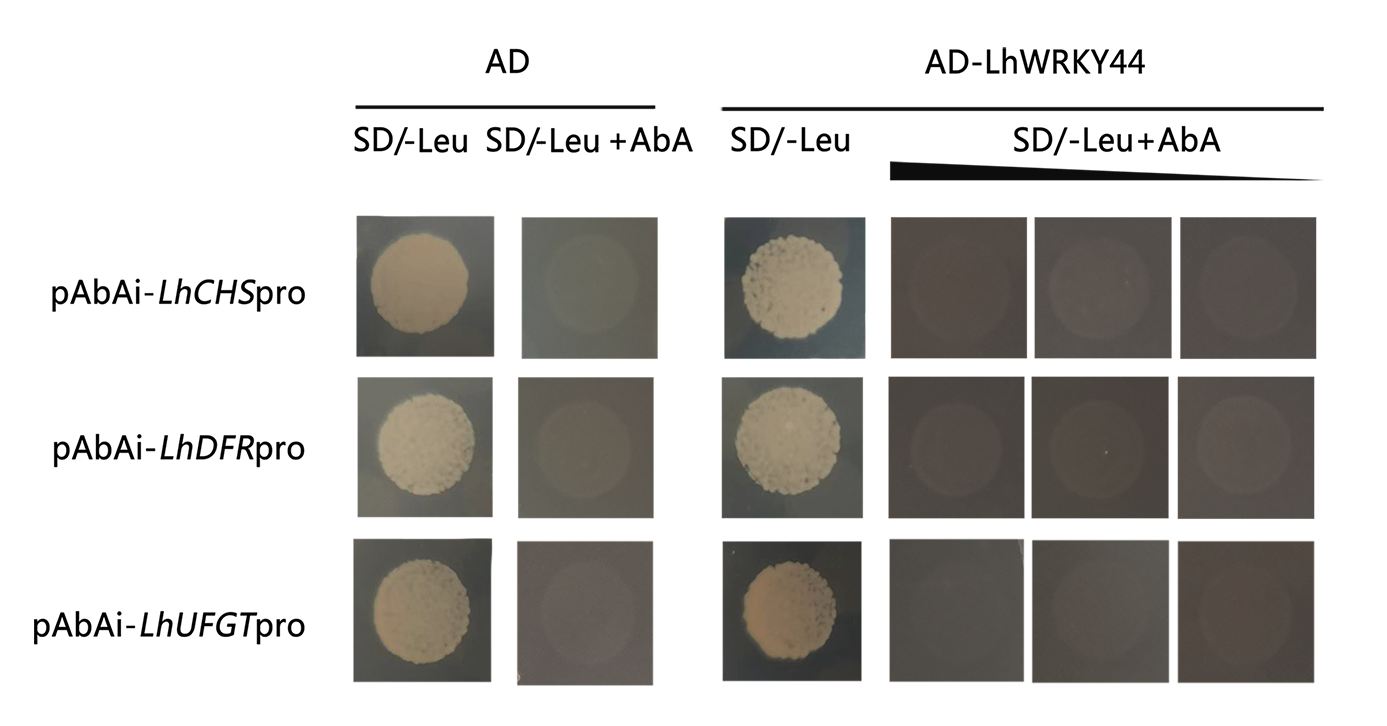

Supplement: Web_Material_uhad167 [file web_material_uhad167.zip › Figure S6.tif]

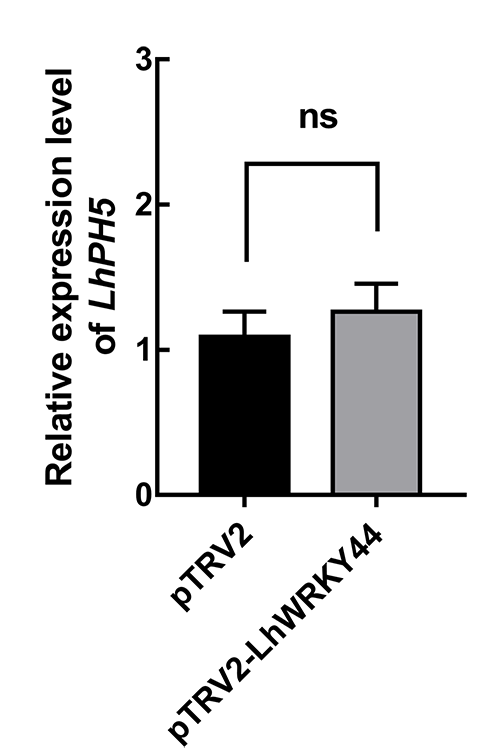

Supplement: Web_Material_uhad167 [file web_material_uhad167.zip › Figure S7.tif]

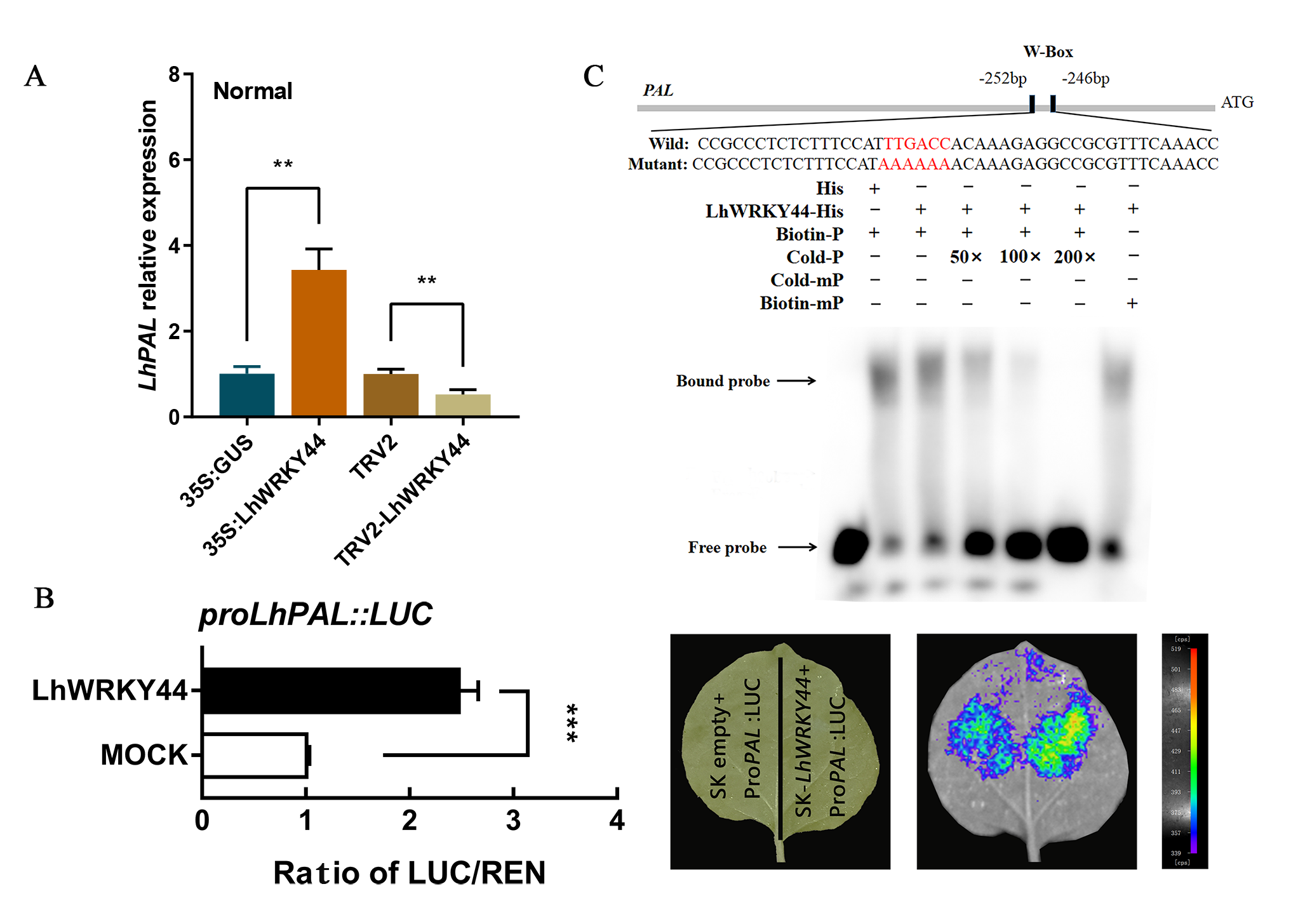

Supplement: Web_Material_uhad167 [file web_material_uhad167.zip › Figure S8.tif]

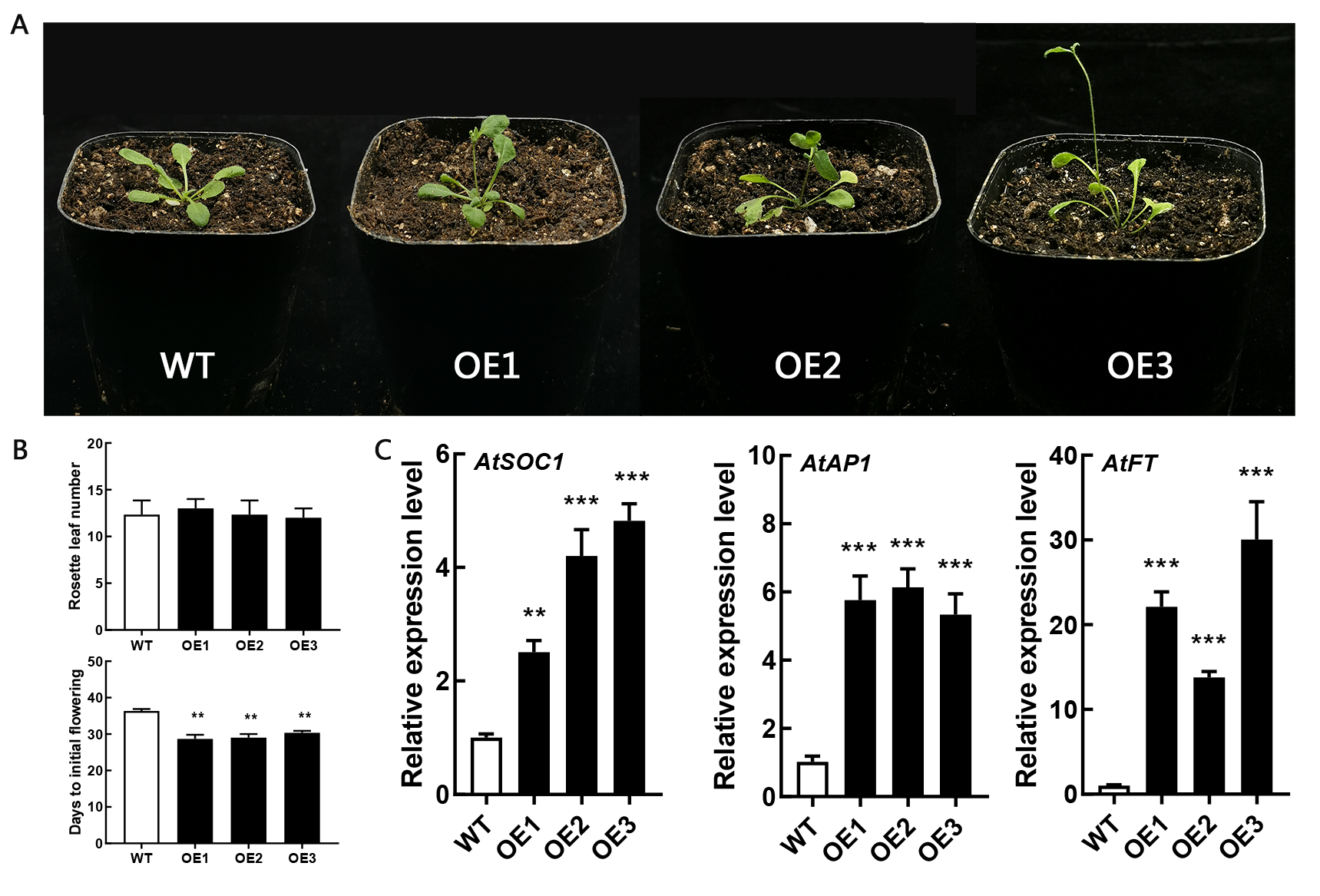

Supplement: Web_Material_uhad167 [file web_material_uhad167.zip › Figure S9.tif]
